# A Cost-Sensitive Behavioral Modeling Analysis of the Early Identification and Control of Infectious Diseases

**DOI:** 10.1007/s11538-026-01696-2

**Published:** 2026-06-26

**Authors:** Mohammad Sharif Ullah, Xiunan Wang, Jin Wang

**Affiliations:** Department of Mathematics, University of Tennessee at Chattanooga, Chattanooga, TN 37403 USA

**Keywords:** Behavioral dynamics, Early diagnosis, False-negative result, Social benefit

## Abstract

A coupled epidemic-behavior model is proposed to assess how vaccination, testing, and treatment jointly influence early outbreak control, accounting for individual choices based on perceived risk, intervention costs, and policy incentives. In a uniformly mixing population, individuals make strategic decisions regarding vaccination and testing participation based on the trade-offs between personal costs and communal benefits, while the intensity of public health testing adjusts according to infection prevalence; the transmission model includes vaccine efficacy, the probability of false negatives in testing, treatment-related reductions in infectious duration and mortality, as well as nonlinear saturation and awareness feedback mechanisms. Analytical threshold results and simulations (time series and two-dimensional parameter sweeps) indicate that reducing vaccination and testing costs expands the disease-free region, while false negatives consistently diminish the efficacy of increased testing by delaying diagnosis and perpetuating onward transmission; treatment reduces transmission potential but maximizes population benefit when combined with prompt implementation of testing. The approach also delineates parameter regimes in which behavioral feedback provides non-monotonic responses and recurring outbreaks, notwithstanding heightened interventions. The findings suggest that effective epidemic preparedness necessitates synchronized incentives for vaccination and testing, investments to minimize false negatives (such as enhanced test accuracy or repeated testing), and treatment accessibility aligned with rapid case identification to optimize resource distribution and early-response strategies during emerging and re-emerging infectious disease threats.

## Introduction

The necessity of pragmatic, adaptable, and scientifically grounded disease control strategies has become increasingly apparent in light of persistent global health challenges, such as the COVID-19 pandemic (Ullah et al. 2021; Derrick et al. 2023) and the emergent and re-emergent infectious diseases (Wang 2022; Ullah and Kabir 2024; Wang and Liao 2012; Bai et al. 2021; Saha et al. 2025; Elsonbaty et al. 2025; Aldila et al. 2024; Soni and Sinha 2024). In this regard, controlling epidemics or pandemics, testing is crucial for promptly diagnosing infected individuals and enabling rapid identification within communities. An effective testing framework detects infected individuals before they undergo extensive transmission, allowing public health officials to implement control strategies such as quarantine, isolation, and treatment. This preventive approach mitigates disease transmission and notifies healthcare systems of impending demand surges, facilitating timely resource distribution. Moreover, comprehensive testing enhances community awareness and encourages individuals to seek treatment, thereby improving the overall public health response and ultimately saving lives. Therefore, traditional disease control methods, which typically rely on fixed-rate testing, standardized vaccination programs, and inflexible treatment protocols, often fall short of effectively controlling rapidly evolving outbreaks in which individual behavior, resource limitations, and pathogen adaptability are crucial factors. This research presents a comprehensive framework that integrates adaptive testing, decision-making based on evolutionary game theory (Tanimoto 2015, 2021), vaccination game dynamics, and treatment interventions to evaluate and enhance disease control dynamics.

Adaptive testing procedures dynamically adjust the pace and emphasis of diagnostic testing based on prevailing epidemiological indices, such as infection prevalence, healthcare burden, and variations in population behavior. These strategies facilitate early diagnosis, optimize isolation protocols, and deploy scarce testing resources more efficiently, reducing disease transmission costs. In contrast, evolutionary game theory-based testing rates simulate strategic interactions among individuals in a population where the prevalence of specific tactics determines payoffs. People must choose among competing methods for infectious diseases, including vaccination, testing, adherence to treatment, or participation in high-risk activities. These judgments are shaped by perceived risks, rewards, and costs, as well as by the behaviors observed in the community. Evolutionary game theory (EGT)- based testing provides (intervention game) a robust framework for analyzing these dynamics, particularly in systems where strategies evolve through imitation, learning, or adaptation. In addition, the vaccination game (Tanimoto 2015, 2021; Ullah et al. 2022; Akter et al. 2024) enhances the EGT model by incorporating the trade-offs people face between vaccination costs and the perceived risk of illness. When people prioritize self-interest, vaccination rates often fall below socially desirable levels, leading to enduring endemic conditions or the potential for outbreaks to recur. Modeling the vaccination choice as a game enables the analysis of equilibrium outcomes and the identification of policy instruments, such as subsidies, fines, or educational initiatives, that steer the population toward more favorable health outcomes. Regardless of individuals’ vaccination status, Chen and Fu (Chen and Fu 2018) use the perception of EGT (Bauch and Earn 2004) through the imitation dynamic (Bauch 2005) to inform individual actions, addressing decision-making obstacles as part of the social-learning process. Fukuda and Tanimoto (Fukuda and Tanimoto 2016) examine the influence of the realities of resolute individuals on others’ vaccination behavior in the spread of infectious diseases within a lattice and a Barabási-Albert scale-free network. Lim and Zhang (Lim and Zhang 2020) employed a nonlinear public goods game to experimentally investigate vaccination decisions. Ida and Tanimoto (Ida and Tanimoto 2017) devised a novel model of a vaccination game to assess the influence of intermediate, noise-perturbing defensive mechanisms in scenarios where players engage freely. Alam et al. (Alam et al. 2018) developed a novel analytical framework using a vaccination game with three or four strategies and an intermediate protective measure. Fu et al. (Fu et al. 2010) employ an EGT framework to investigate the impact of population structure and individual imitative behavior on achieving broad immunity to infectious diseases through voluntary vaccination. Fukuda et al. (Fukuda et al. 2014) assessed the influence of infectious disease risk on individuals' voluntary vaccination behavior within social structures. Bauch and Bhattacharyya (Bauch and Bhattacharyya 2012) scrutinize a model that encapsulates vaccination fear via EGT, using social learning. Kabir et al. (Kabir 2021) presented a vaccination game model to assist policymakers in approving legislation that addresses socioeconomic inadequacy, which is often obscured by complex circumstances.

Furthermore, besides diagnostics and immunization, treatment interventions are crucial for mitigating disease, especially when vaccines are inaccessible or only partially efficacious. The interaction among access to care, personal readiness to seek care, and disease progression profoundly influences the course of an epidemic. Integrating treatment into the comprehensive EGT-based framework enhances our knowledge of the interactions among different control strategies and the impact of people’s strategic decisions on disease transmission and control effectiveness. To address this scenario, Kabir et al. (Kabir et al. 2019) integrate game-theoretic and epidemiological methodologies by combining a disease transmission model with a treatment component and an evolutionary decision-making framework to evaluate behavioral incentives in a vaccination dilemma, in which affected individuals can opt for supplementary treatment. Zobayer et al. (Zobayer et al. 2023) examined the transition rates from susceptible to vaccinated and from infected to treated within a comprehensive evolutionary game-theoretic framework, using a cyclic epidemic model. Poonia and Chakrabarty (Poonia and Chakrabarty 2024) propose a novel two-strain nonlinear mathematical model to assess the impact of treatment availability and adherence on the transmission of human immunodeficiency virus (HIV) within a community. Fitri and Aldila (Fitri and Aldila 2018) assert that both medical treatment and repellent intervention effectively decreased the basic reproduction number, serving as the endemic indication of the model. Gao et al. (Gao et al. 2023) developed a deterministic model to assess how diagnostic testing and compliance with isolation influence the transmission dynamics of COVID-19. In a related direction, Chakraborty et al. (Chakraborty et al. 2025) introduced an extended SEIR framework incorporating testing, treatment, and vaccination, and used evolutionary game theory to examine how human behavioral responses affect the acceptance and implementation of these interventions. More recently, Khatun et al. (Khatun et al. 2026) proposed a behavior-driven epidemic model that couples testing and quarantine decisions with disease transmission, capturing the underlying social dilemma through the concept of social efficiency deficit. These studies collectively show that testing, treatment, vaccination, and compliance behavior can substantially alter epidemic outcomes, and they provide a foundation for incorporating adaptive behavioral mechanisms into intervention-based disease models. Nevertheless, no authors address the important and time-consuming issue of early identification of infected individuals using a testing-based intervention game on a broad scale.

To fill this gap, the current study proposes uncovering the “testing-vaccination-treatment” triad for early disease detection and control, highlighting the influence of each element on human behavior and the spread of infectious individuals. Testing enables prompt detection of infections, thereby reducing the spread of disease by enabling swift intervention. Conversely, vaccination elicits herd immunity, directly reducing the susceptible population. Accessible and effective treatment mitigates the impact of the disease on infected individuals, which is particularly relevant in the modern context, where vaccination skepticism, funding constraints, and rapid pathogen evolution impede disease control efforts. Therefore, the overall goal is to uncover a strategy for policymakers to establish an incentive-related policy to promote socially beneficial behaviors, mitigate the development of resistance, and enhance public health outcomes, offering pragmatic insights for policymakers and health professionals seeking to advance early diagnosis and immunization efforts, thereby strengthening disease resistance at both the individual and societal levels.

## Model and Method

**Fig. 1:**
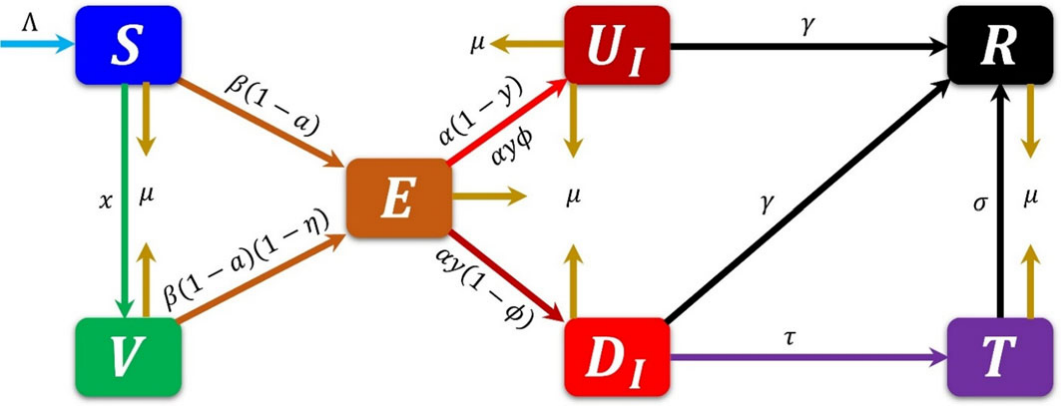
Schematic flow diagram of the model (Color figure online)

A modified SEIR (Ullah et al. 2024; Mohammad et al. 2025) (susceptible-exposed-infected-recovered) epidemic disease model (Fig. 1) is proposed to study the influence of two infection control measures (non-pharmaceutical and pharmaceutical), with testing and vaccination implemented within the game framework. Our proposed model (Fig. 1) consists of seven compartments, representing the respective portions of the total population: susceptible ($S(t)$), vaccinated ($V(t)$), exposed ($E(t)$), undetected infected (${U}_{I}(t)$), detected infected (${D}_{I}(t)$), treated ($T(t)$), and recovered ($R(t)$). Under the assumptions discussed above, the corresponding system of differential Eqs. (1.1–1.7) for the model is as follows:

1 $$\dot{S}=\Lambda -\beta \left(1-a\right)\left({U}_{I}+{D}_{I}\right)S-\left(x+\mu \right)S,$$

1.2 $$\dot{V}=xS-\beta \left(1-\eta \right)\left(1-a\right)\left({U}_{I}+{D}_{I}\right)V-\mu V,$$

1.3 $$\dot{E}=\beta \left(1-a\right)\left({U}_{I}+{D}_{I}\right)S+\beta \left(1-\eta \right)\left(1-a\right)\left({U}_{I}+{D}_{I}\right)V-\left(\alpha +\mu \right)E,$$

1.4 $${\dot{U}}_{I}=\alpha \left(1-y\right)E+\alpha y\phi E-\left(\gamma +\mu \right){U}_{I},$$

1.5 $${\dot{D}}_{I}=\alpha y\left(1-\phi \right)E-\left(\tau +\gamma +\mu \right){D}_{I},$$

1.6 $$\dot{T}=\tau {D}_{I}-\left(\sigma +\mu \right)T,$$

1.7 $$\dot{R}=\gamma \left({U}_{I}+{D}_{I}\right)+\sigma T-\mu R.$$

The total population $N(t)$ is constant, which is scaled to 1:

$$S\left(t\right)+V\left(t\right)+E\left(t\right)+{U}_{I}\left(t\right)+{D}_{I}\left(t\right)+T\left(t\right)+R\left(t\right)=1.$$

Here, susceptible $(S)$ individuals become either alert at a flexible rate $a$ or vaccinated $(V)$ at a rate $x$. Susceptible and vaccinated individuals become exposed $(E)$ (infected in the incubation phase but not infectious) at a rate $\beta$ and $\beta \left(1-\eta \right)$ (lower vaccine efficacy rate) upon contact with other infected individuals. Then, at a constant rate, $\alpha$ exposed individuals become infected, and they are identified $({D}_{I})$ by testing rate $\alpha y$, and the remaining fraction of infected individuals stays with the population, i.e., not identified $({U}_{I})$ by rate $\alpha \left(1-y\right)$. During the testing time, some of the infected individuals get false negative results and remain in ${U}_{I}$ class at the rate $\alpha y\phi .$ Infected individuals who are detected join the treated class $(T)$ at the rate $\tau$. Infected (detected and undetected) recover $(R)$ at a rate $\gamma$. Natural birth and death rates are $\Lambda$ and $\mu$ (for more details, see Table 1).

**Table 1:** List of parameters, variables, and their biological meanings

Notation	Meaning	Value	Reference
$\Lambda$	Birth rate	–	Estimated
$\beta$	Transmission rate	0.8333	Zobayer et al. 2023)
$a$	Personal awareness	0.1–0.9	Akter et al. 2024)
$\mu$	Death rate	–	Estimated
$\alpha$	Incubation period	0.2	Zobayer et al. 2023)
$\gamma$	Recovery rate	0.1	Estimated
$y$	Testing rate	0.1–0.9	Estimated
$x$	Vaccination rate	0.01–0.1	Zobayer et al. 2023)
$\tau$	Detected people’s treatment rate	0.9	Estimated
$\sigma$	Treatment recovery rate	0.2	Zobayer et al. 2023)
${m}_{x}$	Vaccination uptake rate	0.1	Akter et al. 2024)
${m}_{y}$	Testing uptake rate	0.1	Estimated
${C}_{y}$	Cost of testing	0.1–0.9	Estimated
${C}_{I}$	Cost of infection	1.0	Akter et al. 2024)
${C}_{V}$	Cost of vaccine	0.1–0.9	Zobayer et al. 2023)
$\phi$	The false negative result rate of testing	0.0–0.4	Estimated
$\delta$	Controlling parameter (EGT)	0.1	Estimated
$\lambda$	Controlling testing rate (Adaptive)	0.1–0.9	Estimated
$k$	Saturation rate	0.1–0.9	Estimated
${B}_{V}$	Benefit rate of vaccination	0.1–0.9	Estimated
$\omega$	Hesitancy rate of vaccination	0.0–0.4	Estimated
${y}_{M}$	Motivation for testing	0.1–0.9	Estimated

Furthermore, in our model, awareness denotes an individual’s perception of infection risk and severity that prompts protective behavioral modifications, such as minimizing close contacts, enhancing hygiene, utilizing masks, avoiding crowded environments, or self-isolating when symptomatic; it is implicitly represented through its influence on transmission rather than as a distinct compartment. The parameter $a$ represents the personal protection rate, specifically an awareness-induced protection level, a dimensionless variable constrained to $0\le a\le 1$, which quantifies the efficacy of awareness in translating into preventive measures that mitigate infection risk. Mathematically, influences the force of infection via the factor $(1-a)$, resulting in effective transmission defined as $\beta (1-a)({U}_{I}+{D}_{I})$. Here, $a=0$ signifies no additional protection beyond baseline behavior, while higher values of $a$ denote increased adherence to awareness or personal protection, thereby proportionately reducing the rate at which susceptible and vaccinated individuals contract the infection.

### Model Limitations

A key constraint of the proposed framework is the assumption of homogeneous mixing, which posits that all individuals are equally likely to interact, thereby failing to capture the realistic heterogeneity in contact patterns, clustering, age demographics, household transmission, or context-specific exposure (such as in schools, workplaces, and healthcare facilities). Correspondingly, behavior is encapsulated by population-level strategy shares (e.g., testing participation $y(t)$ and protection level $a$), the model fails to account for individual-level discrepancies in risk perception, access impediments, misinformation, or compliance trajectories, nor does it explicitly integrate behavioral fatigue, temporal fluctuations in trust, or delayed reactions to incidence. A false-negative mechanism and adaptive intensity characterize the testing process, yet it neglects operational constraints such as limited test availability, turnaround delays, targeted testing of high-risk populations, and the potential for false positives; likewise, treatment is depicted through average reductions in infectious duration and mortality instead of capacity limitations, triage, or adherence issues. Ultimately, parameters such as perceived costs and incentives are challenging to quantify directly and vary across contexts; thus, quantitative forecasts should be regarded as scenario-based insights. Enhancements through structured mixing (age/degree classifications), spatial or network models, and data-informed behavioral calibration would bolster external validity.

### Behavioral Dynamics of Evolutionary Game Theory (EGT)

Individuals can choose vaccinations and testing programs based on their interests and strategies by observing the number of people infected at any given time during a single season. In the behavioral dynamics, each participant can choose to participate in the vaccine program or not, depending on the vaccine cost and associated factors, as well as whether to adopt a proactive intervention. Furthermore, if infected individuals wish to test themselves, a retroactive provision allows them to compare their strategies based on recovery time, testing costs, and disease incidence. Thus, if the individual becomes vaccinated and tested at a vaccination rate $(x)$ and a testing rate $(y)$, the equation that describes the human behavioral dynamics is,

2.1 $$\dot{x}={m}_{x}x\left(1-x\right)\left[-{C}_{V}V+{C}_{I}\left({U}_{I}+{D}_{I}\right)+{B}_{V}-\omega S\right].$$

$$\dot{y}(\mathbf{b}\mathbf{e}\mathbf{h}\mathbf{a}\mathbf{v}\mathbf{i}\mathbf{o}\mathbf{r}\mathbf{a}\mathbf{l}\,\mathbf{t}\mathbf{e}\mathbf{s}\mathbf{t}\mathbf{i}\mathbf{n}\mathbf{g}\,\mathbf{r}\mathbf{a}\mathbf{t}\mathbf{e})={m}_{y}y\left(1-y\right)\left[-(1-{C}_{y})\delta +{C}_{I}\left({U}_{I}+{D}_{I}\right)+{y}_{M}\right]$$. (2.2).

Here, ${C}_{V}$ and ${C}_{y}$ are the relative costs of vaccination and testing as compared with the disease cost, ${C}_{I} (=1)$. The payoff difference (gain) between cooperation and defection, [$-{C}_{V}V+{C}_{I}\left({U}_{I}+{D}_{I}\right)+{B}_{V}-\omega S$], whether positive or negative, determines the vaccination decision in Eq. (2.1). In Eq. (2.2), the payoff gain for testing methods [$-(1-{C}_{y})\delta +{C}_{I}\left({U}_{I}+{D}_{I}\right)+{y}_{M}$] is more favorable than testing for disease duration. Also, the motivation rate ${y}_{M}$ is an exogenous (baseline) drive that enhances an individual's propensity to engage in testing, irrespective of the prevailing epidemic or pandemic payoff indications. It signifies enduring factors such as public health communications, reminders and outreach efforts, societal norms, institutional mandates, accessibility (proximity of locations, minimal waiting periods), and overall health-seeking behaviors that maintain the appeal of testing despite a diminished perception of infection risk. Mathematically, ${y}_{M}$ serves as a continuous, positive incentive in the payoff term, directing the replicator dynamics toward increased testing participation ($y(t)$) and helping prevent a decline in testing uptake during periods of low prevalence.

### Adaptive Testing Rate with Saturation

The adaptive testing rate with saturation is crucial for epidemic disease control, as it enables dynamic adjustment of testing intensity based on the outbreak’s current status while accounting for practical constraints. Testing resources, including laboratory capacity, medical personnel, and diagnostic supplies, are limited in practical contexts. Therefore, although testing should ideally increase as infection levels rise, it cannot expand indefinitely. Saturated adaptive testing rates reflect this phenomenon by permitting the testing rate to increase with infection prevalence, stabilizing as resource limitations are encountered, thereby enhancing the accuracy of predictions of epidemic dynamics, optimizing the timing and targeting of interventions, and underscoring the need to bolster health system capacities. The integration of adaptive saturation in testing rates enhances comprehension of the impact of resource constraints on the effectiveness of testing-based control strategies, thereby facilitating the formulation of robust public health responses that align demand with available resources. Thus, the **adaptive testing rate**:

2.3 $$\dot{y}\left(\mathbf{a}\mathbf{d}\mathbf{a}\mathbf{p}\mathbf{t}\mathbf{i}\mathbf{v}\mathbf{e}\,\mathbf{t}\mathbf{e}\mathbf{s}\mathbf{t}\mathbf{i}\mathbf{n}\mathbf{g}\,\mathbf{r}\mathbf{a}\mathbf{t}\mathbf{e}\right)=-{C}_{y}y+\frac{\lambda \left({U}_{I}+{D}_{I}\right)}{1+ky}.$$

Here, ${C}_{y}$ is the relative cost of testing, $\lambda$ is the testing control rate, and $k$ is the saturation rate.

#### The Partial Rank Correlation Coefficients (PRCCs)

**Fig. 2:**
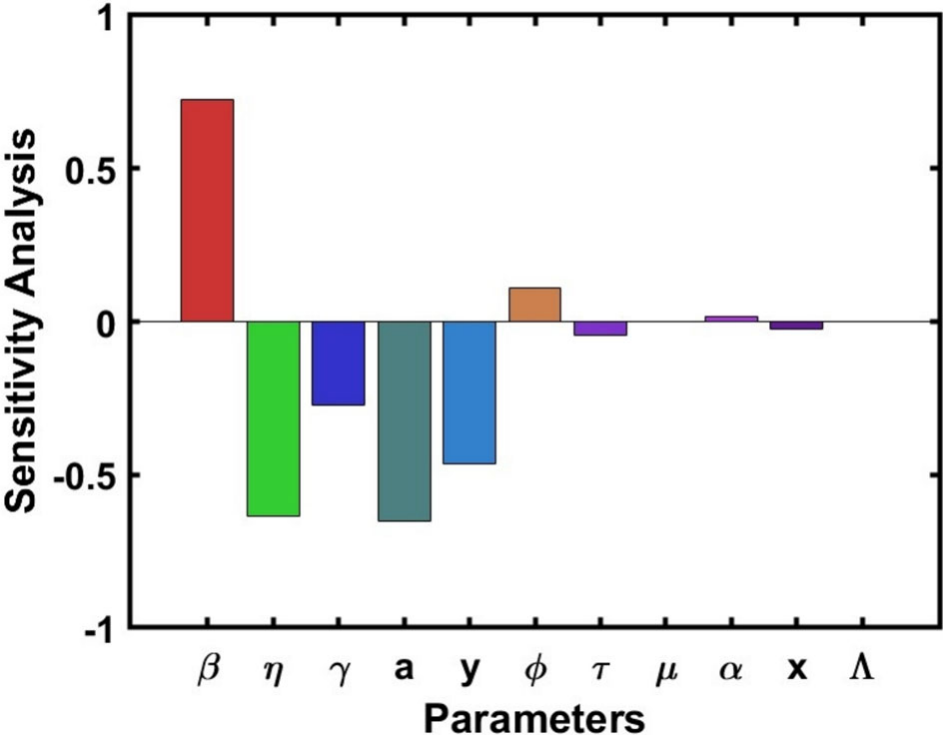
Partial rank correlation coefficients (PRCCs) and the sensitivity index of various parameters (spanning from $-1$ to $1$) are concerned with ${R}_{0}$ only (Color figure online)

Figure 2 depicts a Partial Rank Correlation Coefficient (PRCC) sensitivity analysis (Ullah et al. 2024) plot that evaluates the impact of fluctuations in crucial epidemiological and behavioral factors on the basic reproduction number ${R}_{0}$ for the model with constant parameters [see Eq. (5) in the Appendix]. The horizontal axis denotes the investigated parameters: $\beta$ (transmission rate), $\eta$ (vaccine effectiveness), $\gamma$ (recovery rate), $a$ (awareness rate), $y$ (testing rate), $\phi$ (false-negative result rate), and $\tau$ (treatment rate). The vertical axis measures the magnitude and orientation of each parameter’s influence on ${R}_{0}$, spanning from $-1$ to $1$.

The bar representing $\beta$ is elevated and positive, indicating a strong positive correlation with ${R}_{0}$; when the transmission rate heightens, ${R}_{0}$ markedly upsurges. The awareness rate $(a)$, testing rate $(y)$, and vaccination effectiveness $(\eta )$ exhibit moderate to strong negative correlations, indicating that improvements in public awareness, testing initiatives, or vaccine efficacy significantly reduce disease transmission. The parameters $\gamma$ (recovery rate) and $\tau$ (treatment rate) have mild negative correlations, demonstrating a very negligible impact on ${R}_{0}$. Notably, $\phi$ (false-negative rate) has a slight positive influence, suggesting that inadequate test accuracy marginally exacerbates epidemic consequences. In recapitulation, Fig. 2 accentuates that regulating transmission ($\beta$) and improving awareness ($a$), testing ($y$), and vaccination effectiveness ($\eta$) are the most essential factors in diminishing the basic reproduction number and controlling the epidemic’s dissemination. Table 2 outlines critical parameters in our proposed model, including transmission $(\beta )$, vaccine efficacy $(\eta )$, recovery $(\gamma )$, and personal awareness $\left(a\right),$ testing $(y),$ false negative result $(\phi ),$ treatment $(\tau )$, death $(\mu )$, incubation $(\alpha )$, vaccination $(x)$, and birth $(\Lambda )$ rates.

**Table 2:** The sensitivity index of key parameters

Parameter	Sensitivity index
$\beta$	+ 0.724112
$\eta$	− 0.634428
$\gamma$	− 0.292413
$a$	− 0.650316
$y$	− 0.462957
$\phi$	+ 0.116521
$\tau$	− 0.0426301
$\mu$	0.0
$\alpha$	+ 0.017629
$x$	− 0.023511
$\Lambda$	0.0

### ***Box Plot Analysis Of The Basic Reproduction Number***${{\boldsymbol{R}}}_{0}$

**Fig. 3:**
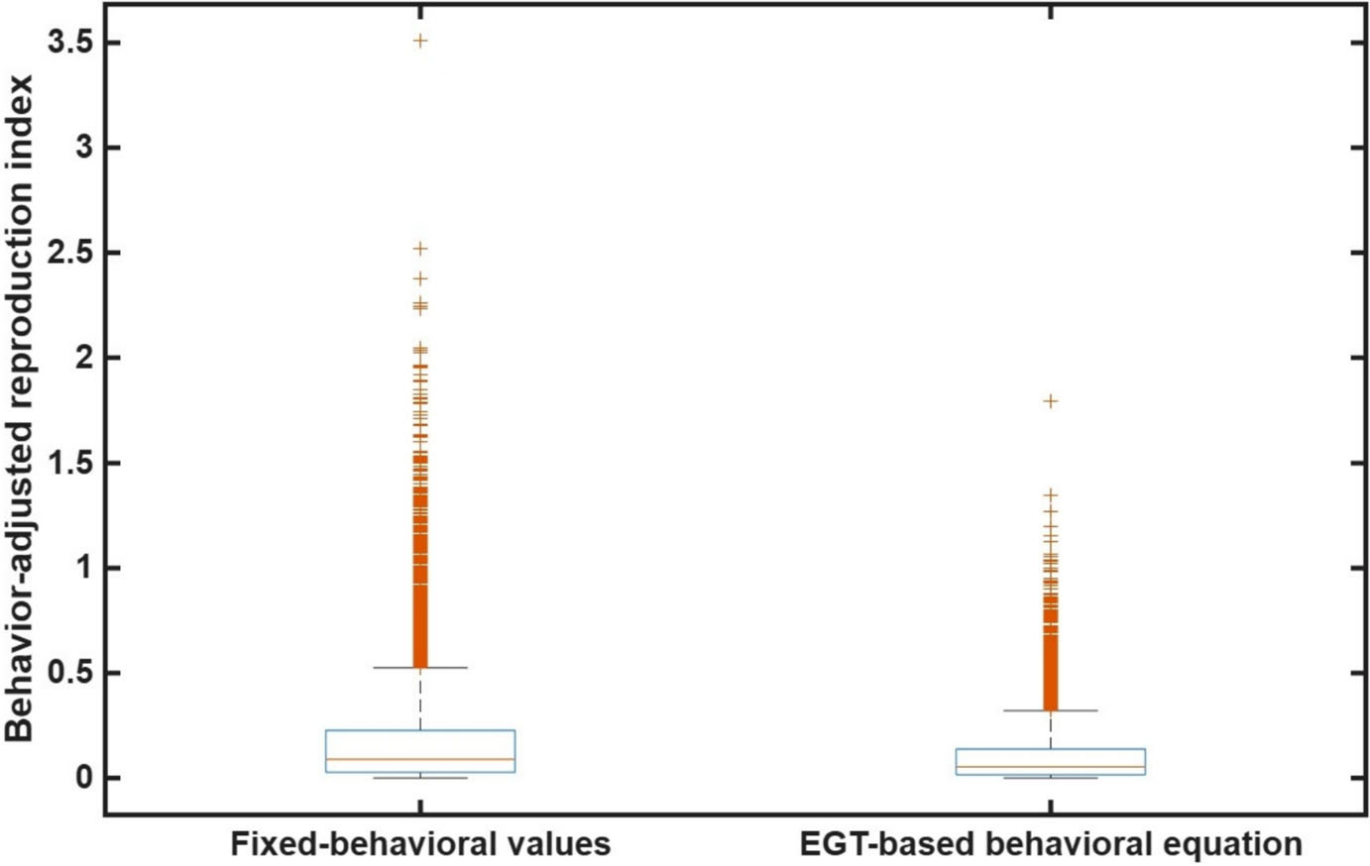
Box counting dimension of ${R}_{0}$ concerning analytical formulation and the epidemic model with EGT-based vaccination and testing rates (Color figure online)

Figure 3 compares the distribution of the reproduction-related threshold quantity under two behavioral assumptions: fixed behavior (the analytical formulation of ${R}_{0}$ presented Eq. (5)) and EGT-based adaptive behavior (Eqs. (1.1–1.7) with (2.1–2.2), the full epidemic model with behavioral equations of vaccination and testing rates), where the parameters were generated randomly **(**10,000 Monte Carlo simulations) but systematically; each parameter was sampled independently from a predefined biologically admissible uniform range. In the fixed-behavior case, all parameters and the behavioral variables $x$ and $y$ were randomly sampled from prescribed intervals, $x\in [\mathrm{0.01,0.05}]$ and $y\in [\mathrm{0.01,1.0}]$, and then held constant during the threshold calculation. In the EGT-based case, the same sampled initial behavioral values were used, but the full epidemic-behavior model was simulated so that $x(t)$ and $y(t)$ evolved according to the EGT equations before the threshold quantity was evaluated. Here, the fixed-behavioral value boxplot shows a larger median, a wider interquartile range, and a heavier upper tail, indicating that when vaccination and testing rates are fixed (forced), the transmission potential remains more sensitive to unfavorable parameter combinations. Several upper outliers appear, reaching values above ${R}_{0}=3$, which reflects parameter sets in which transmission intensity, weak protection, or low behavioral response allows the threshold quantity to become large. In contrast, the EGT-based behavior boxplot is shifted downward and is more concentrated near lower values. Its median and interquartile range are smaller, and the upper outliers are less extreme than in the fixed-behavioral value case, indicating that adaptive behavioral feedback reduces both the average transmission potential and its variability. Realistically, once vaccination and testing/treatment-related behavior are allowed to adjust dynamically in response to epidemic conditions, the model produces lower, more stable threshold values across the sampled parameter space.

## Results and Discussion

This section describes disease dynamics in detail using a line graph and a 2D heatmap. To generate these results, the well-known finite difference scheme is used. It is worth noting that for other numerical simulations, such as generating line graphs and 2D heatmaps, population birth and death rates are not considered, as our study focuses on a relatively short time frame and natural birth and death rates are negligible.

**Fig. 4:**
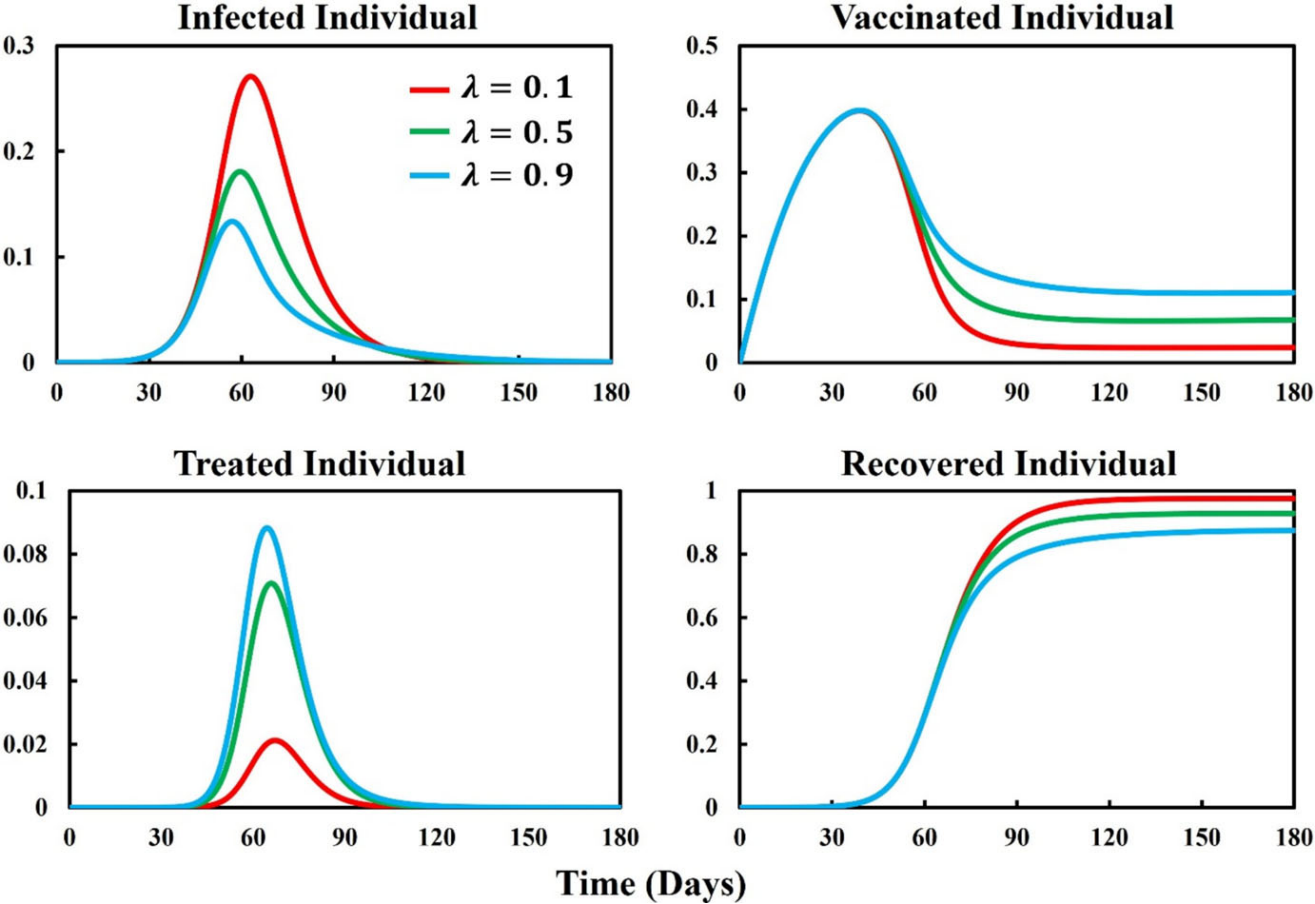
The effect of the controlling testing rate $(\lambda =\mathrm{0.1,0.5,0.9})$ on the infected, vaccinated, treated, and recovered individuals through adaptive testing and EGT-based vaccination rates. The parameter settings are $\beta =0.8333,\gamma =0.1,\alpha =0.2,\phi =0.05,a=0.2,\tau =0.9,\sigma =0.2,\omega =0.2,{m}_{x}=0.1,{C}_{V}=0.5,\eta =0.5,{C}_{y}=0.1,k=0.1,{B}_{V}=0.2$ (Color figure online)

Figure 4 illustrates the impact of controlling the testing process during a pandemic or epidemic. The regulation of testing rates is crucial in shaping the dynamics of infected, vaccinated, treated, and recovered individuals within a community, particularly when EGT informs adaptive testing procedures and vaccination rates. By adapting the testing rate to real-time infection data and behavioral responses, public health systems can more efficiently detect and isolate affected individuals, thereby mitigating infection transmission (Fig. 4, infected curve). This focused detection enables prompt treatment, thereby enhancing recovery rates and mitigating illness severity. In real-world phenomena, when people observe that the government or private health sector works effectively to combat disease and takes necessary actions to reduce the severity of the disease for infected individuals who are undergoing treatment (Fig. 4, treated (blue) curve), they become motivated to receive necessary treatments and recover from the disease. In addition, knowledge of testing often impacts personal behavior, including the propensity to vaccinate (Fig. 4, vaccinated (blue) curve), particularly when vaccination choices are analyzed through EGT. Within these frameworks, people assess the perceived costs and benefits of vaccination based on the observed disease prevalence and their peers’ behavior. Consequently, an increased adaptive testing rate indirectly enhances vaccination rates by strengthening the perceived risk of infection. This deliberate interaction between testing and vaccination accelerates the transition of individuals from the infected state to the treated and recovered categories, ultimately flattening the epidemic curve and enhancing public health outcomes.

**Fig. 5:**
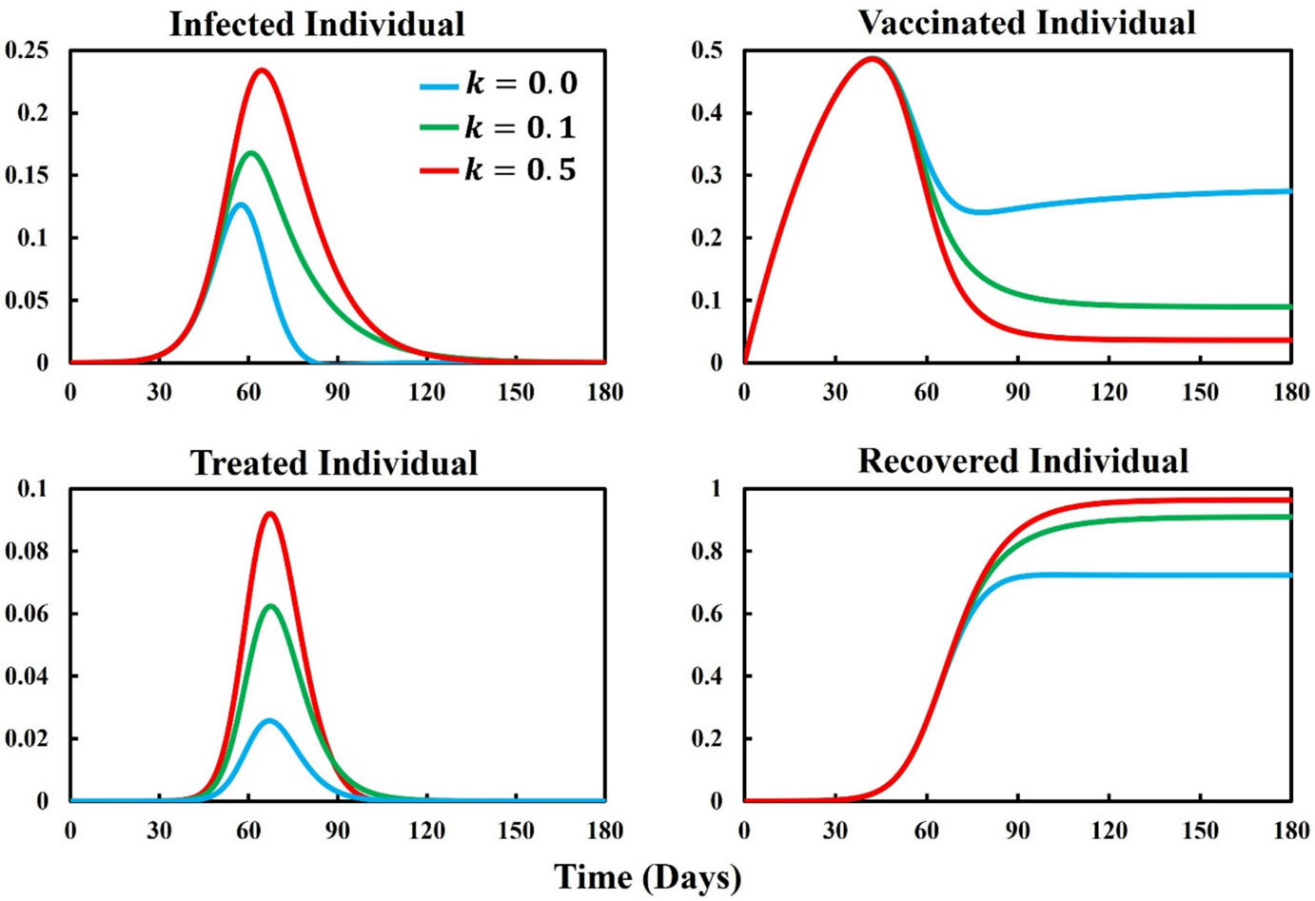
The effect of the saturation $(k=\mathrm{0.0,0.1,0.5})$ on the infected, vaccinated, treated, and recovered individuals through adaptive testing rates. The parameter settings are $\beta =0.8333,\gamma =0.1,\alpha =0.2,\phi =0.05,a=0.2,\tau =0.9,\sigma =0.2,\omega =0.2,{m}_{x}=0.1,{C}_{V}=0.5,\eta =0.5,{C}_{y}=0.1,\lambda =0.1,{B}_{V}=0.2$ (Color figure online)

The saturation strategy for testing rates, in which testing capacity reaches its limit, has a profound impact on disease dynamics, particularly when combined with adaptive testing and EGT-based immunization regimens, as depicted in Fig. 5. As testing rates approach saturation, the capacity to detect and isolate infected individuals diminishes, undermining the efficacy of adaptive testing in mitigating transmission, a severe outcome for any society. This bottleneck leads to an undercount of current cases, which diminishes the perceived urgency of the pandemic or epidemic and, consequently, reduces public adherence to health protocols. If testing for saturation obscures the true incidence of the illness, people may underestimate their risk and decline to participate in immunization programs, which, in turn, diminishes overall vaccine coverage. In contrast, when testing is adaptive and responsive, without reaching saturation, it delivers prompt, precise feedback on infection rates, thereby promoting higher vaccination rates and more proactive treatment-seeking behavior. Consequently, preventing saturation in testing capacity is essential for preserving the integrity of adaptive treatments, ensuring a consistent public response, facilitating a more rapid transition from susceptible to vaccinated or recovered classes, and enhancing epidemic control, a crucial strategy for policymakers to mitigate the disease in society.

**Fig. 6:**
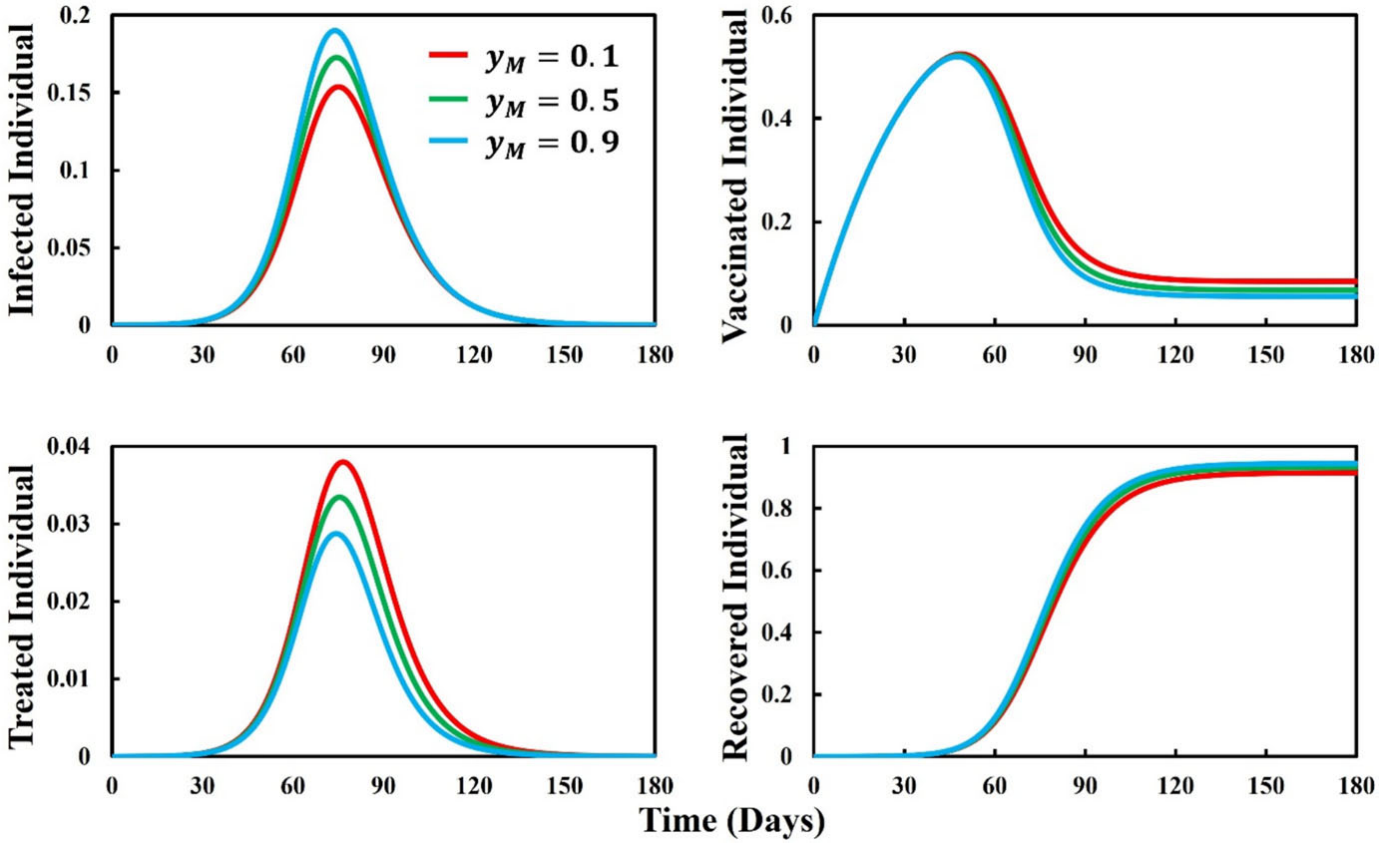
The effect of the testing awareness or motivation program $({y}_{M}=\mathrm{0.1,0.5,0.9})$ on the infected, vaccinated, treated, and recovered individuals through EGT-based testing and vaccination rates. The parameter settings are $\beta =0.8333,\gamma =0.1,\alpha =0.2,\phi =0.05,a=0.2,\tau =0.9,\sigma =0.2,\omega =0.2,{m}_{x}=0.1,{C}_{V}=0.5,\eta =0.5,{C}_{y}=0.1,\delta =0.1,{B}_{V}=0.2$ (Color figure online)

Figure 6 illustrates the impact of motivation programs and government actions on participation in testing programs during pandemics or epidemics, particularly for individuals exposed to the disease or those in contact with infected individuals, as awareness and motivation programs have a significant impact on disease dynamics by enhancing people’s responses to testing and vaccine choices, particularly when the EGT mechanism analyzes these behaviors. Such initiatives raise public awareness of the benefits of early diagnosis and prevention, encouraging people to test more frequently and consistently. Within an EGT paradigm, as more individuals participate in testing driven by heightened desire, identifying asymptomatic or early-stage diseases becomes easier, facilitating prompt isolation and referral for appropriate treatment, thereby reducing disease severity and transmission. The increased awareness of the pandemic alters people’s risk perception, often leading to a heightened inclination to vaccinate as a preventive measure. Thus, the testing awareness initiative establishes a feedback loop: enhanced testing reveals more illnesses, heightening perceived risk and improving vaccination rates. This collective behavioral change, driven by knowledge and adaptive decision-making, leads to lower infection rates, higher treatment and recovery rates, and more effective public health responses.

**Fig. 7:**
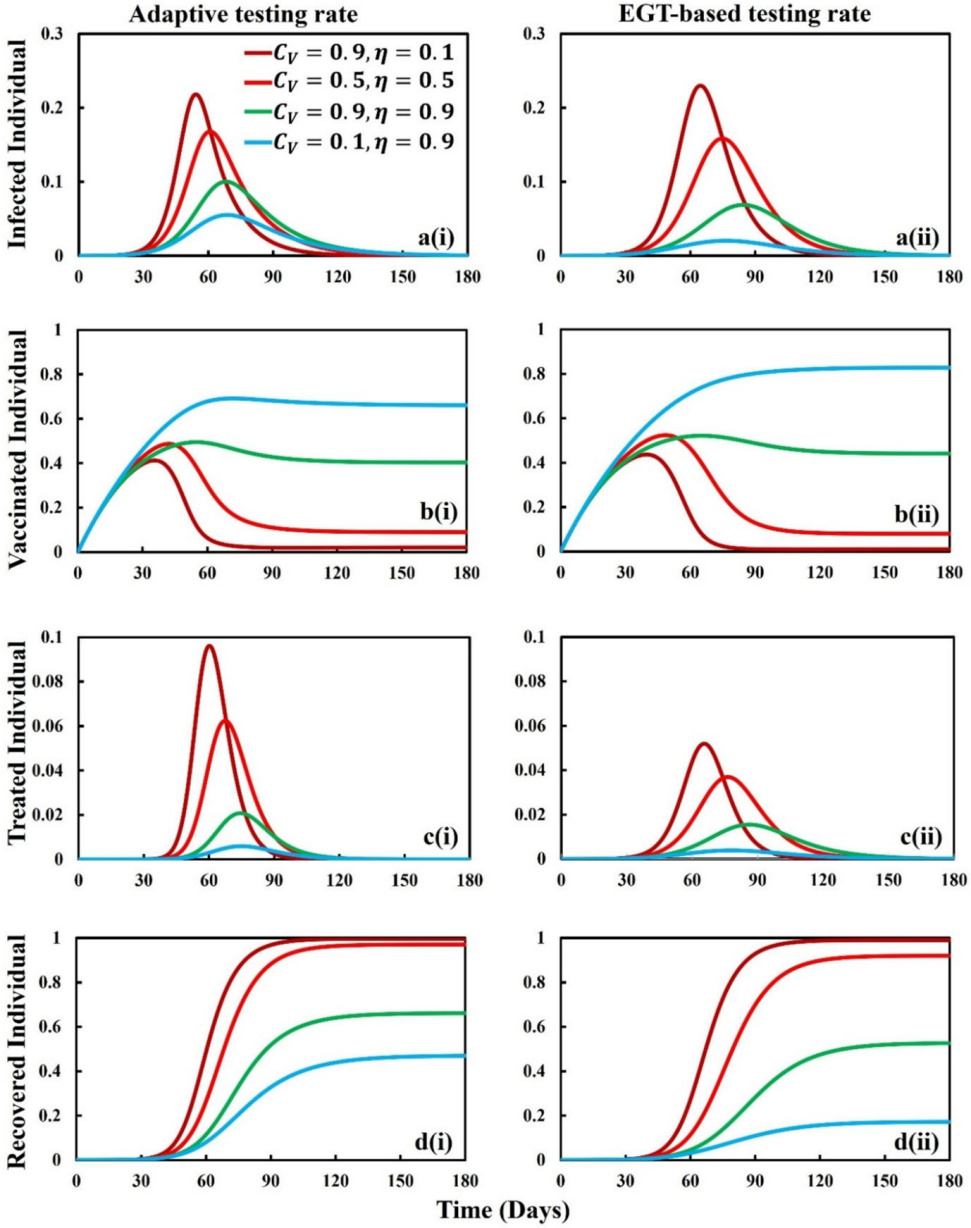
The effect of vaccination cost and efficacy $(\left({C}_{V},\eta \right)=\left(\mathrm{0.9,0.1}\right),\left(\mathrm{0.5,0.5}\right),\left(\mathrm{0.9,0.9}\right),\left(\mathrm{0.1,0.9}\right))$ on the infected, vaccinated, treated, and recovered individuals through adaptive (Panel i*-) and EGT-based (Panel ii*-) testing rates. The parameter settings are $\beta =0.8333,\gamma =0.1,\alpha =0.2,\phi =0.05,a=0.2,\tau =0.9,\sigma =0.2,\omega =0.2,{m}_{x}={m}_{y}=0.1,{B}_{V}=0.2,{C}_{y}=0.1.$ For adaptive testing case, $k=0.1,\lambda =0.1,$ and EGT case, ${y}_{M}=0.2,\delta =0.1$ (Color figure online)

The cost $({C}_{V})$ and the effectiveness of vaccination (η) are critical elements influencing disease dynamics, particularly when combined with testing and vaccination techniques based on an adaptive and EGT framework, as illustrated in Fig. 7. When vaccine effectiveness is high and costs are low (Fig. 7, blue curve), the perceived benefits of vaccination increase, leading to higher vaccination rates. In turn, this reduces the vulnerable population and enhances herd immunity, resulting in lower infection rates and fewer people requiring treatment. In contrast, elevated vaccination costs or diminished vaccine effectiveness diminish individual motivation to vaccinate, undermining overall vaccine coverage and facilitating persistent or recurrent transmission (Fig. 7, deep red curve). In addition, adaptive and EGT-based testing rates further influence these processes. Frequent, responsive testing, informed by real-time data and EGT-modelled individual choices, improves understanding of infection risks. Despite its disadvantages, the heightened perceived danger partly counterbalances the deterrent impact of elevated vaccination costs or diminished efficiency by incentivizing vaccination as a preventative measure. In summary, extensive testing facilitates early identification and transfer of isolated individuals for treatment, aiding epidemic control even when vaccination rates are inadequate. The interplay between vaccine cost and effectiveness, influenced by adaptive testing and behaviorally responsive (EGT-based) techniques, is essential in shaping the overall course of an epidemic. Economical, highly efficient vaccinations and robust testing policies provide ideal circumstances for rapid and sustained disease control.

Furthermore, as shown in Fig. 7, infected, vaccinated, and treated outcomes reduce infection incidence and vaccination during adaptive testing. Nevertheless, the number of treated persons is higher than with EGT-based testing because the distinct processes that influence testing behavior differ between the methodologies. The primary reason is that adaptive testing is often guided by real-time epidemiological data or by governmental/public health interventions that aim to enhance testing in response to dynamically escalating infection rates. This proactive strategy facilitates earlier and more efficient detection and treatment of sick individuals, even with modest overall vaccination rates. Consequently, more individuals enter the treated compartment before the progression or further dissemination of the illness, resulting in an elevated treated population curve. Moreover, due to prompt disease detection and efficient control, the incidence of infection has been reduced. As EGT-based testing relies on individuals’ strategic decision-making, this decentralized, reactive approach often leads to delays or discrepancies in testing, particularly when the perceived risk of infection is low or when testing is considered burdensome or unnecessary. Consequently, fewer cases will be identified and addressed promptly, resulting in a reduced treatment curve and potentially elevated infection rates. Moreover, individuals within EGT-based frameworks exhibit a heightened response to observable danger, which may lead to increased vaccination rates during disease surges, contributing to the relatively elevated vaccination rate. Additionally, adaptive testing achieves a superior treated population by emphasizing rapid and uniform case detection and response. In contrast, EGT-based testing exhibits significant variability and higher reliance on individual perception, thereby hindering diagnosis and treatment.

**Fig. 8:**
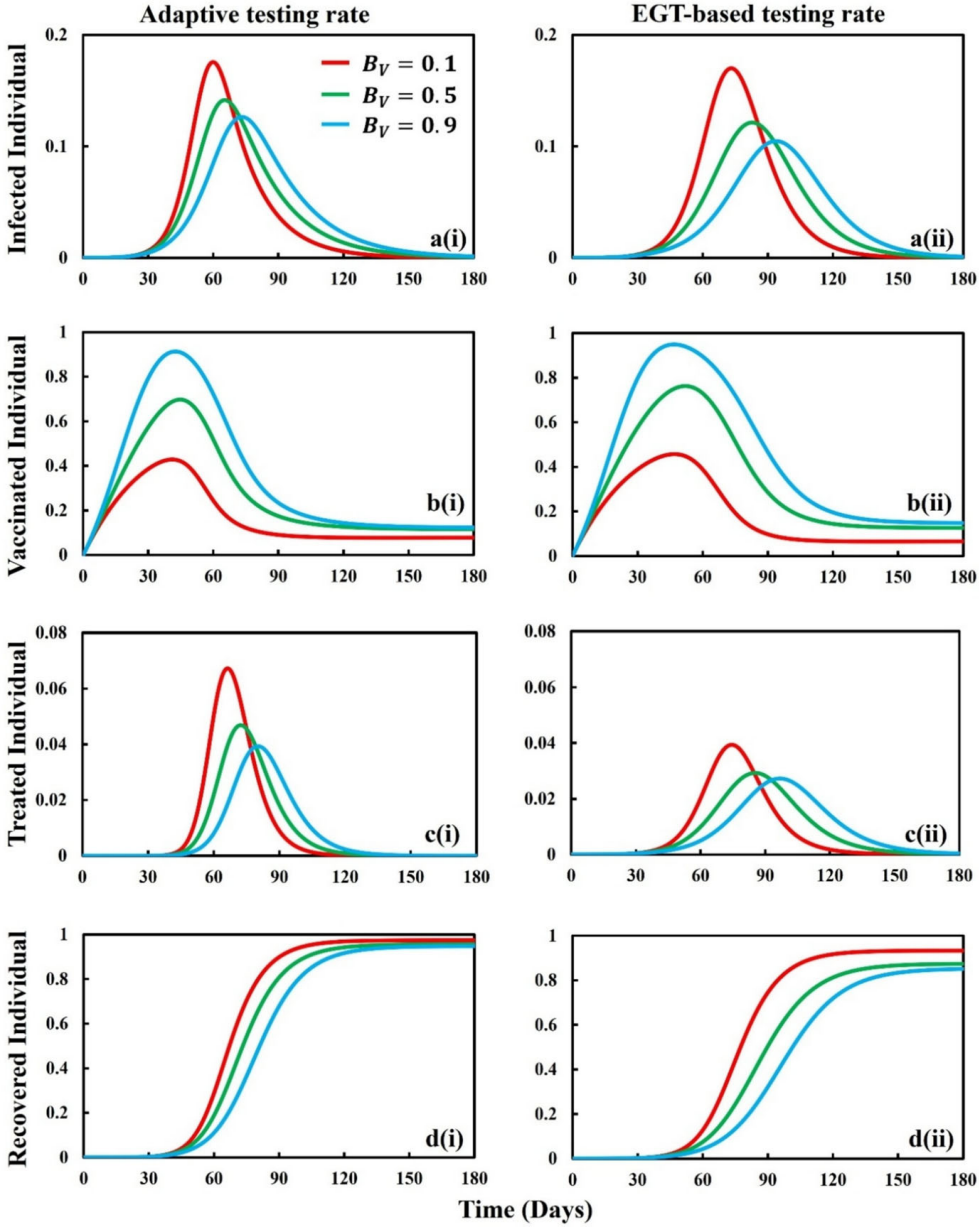
The effect of the benefits of vaccination $({B}_{V}=\mathrm{0.1,0.5,0.9})$ on the infected, vaccinated, treated, and recovered individuals through adaptive (panel i*-) and EGT-based (panel ii*-) testing rates. The parameter settings are $\beta =0.8333,\gamma =0.1,\alpha =0.2,\phi =0.05,a=0.2,\tau =0.9,\sigma =0.2,\omega =0.2,{m}_{x}={m}_{y}=0.1,{C}_{V}=0.5,\eta =0.5,{C}_{y}=0.1.$ For adaptive testing case, $k=0.1,\lambda =0.1,$ and EGT case, ${y}_{M}=0.2,\delta =0.1$ (Color figure online)

The perceived advantages of vaccination significantly influence disease trends, particularly when vaccination rates are facilitated by adaptive and EGT-based approaches, as shown in Fig. 8. When vaccination is highly advantageous due to its robust effectiveness, few side effects, and social or economic incentives, individuals are more likely to choose vaccination within the EGT framework. The heightened adoption reduces vulnerability and mitigates disease transmission. Moreover, adaptive testing enhances public awareness and perceived risk, increasing the appeal of vaccination as a preventive intervention. In EGT-based testing, the visibility of extensive vaccination may modify societal norms and alter cost–benefit perceptions, thereby indirectly encouraging further testing and vaccination. As vaccination coverage increases due to these perceived advantages, disease incidence declines, alleviating strain on healthcare systems and shortening outbreak duration. Enhancing vaccine advantages establishes a positive feedback loop between individual choices and adaptive public health measures, eventually reducing transmission rates and facilitating faster recovery at the community level.

**Fig. 9:**
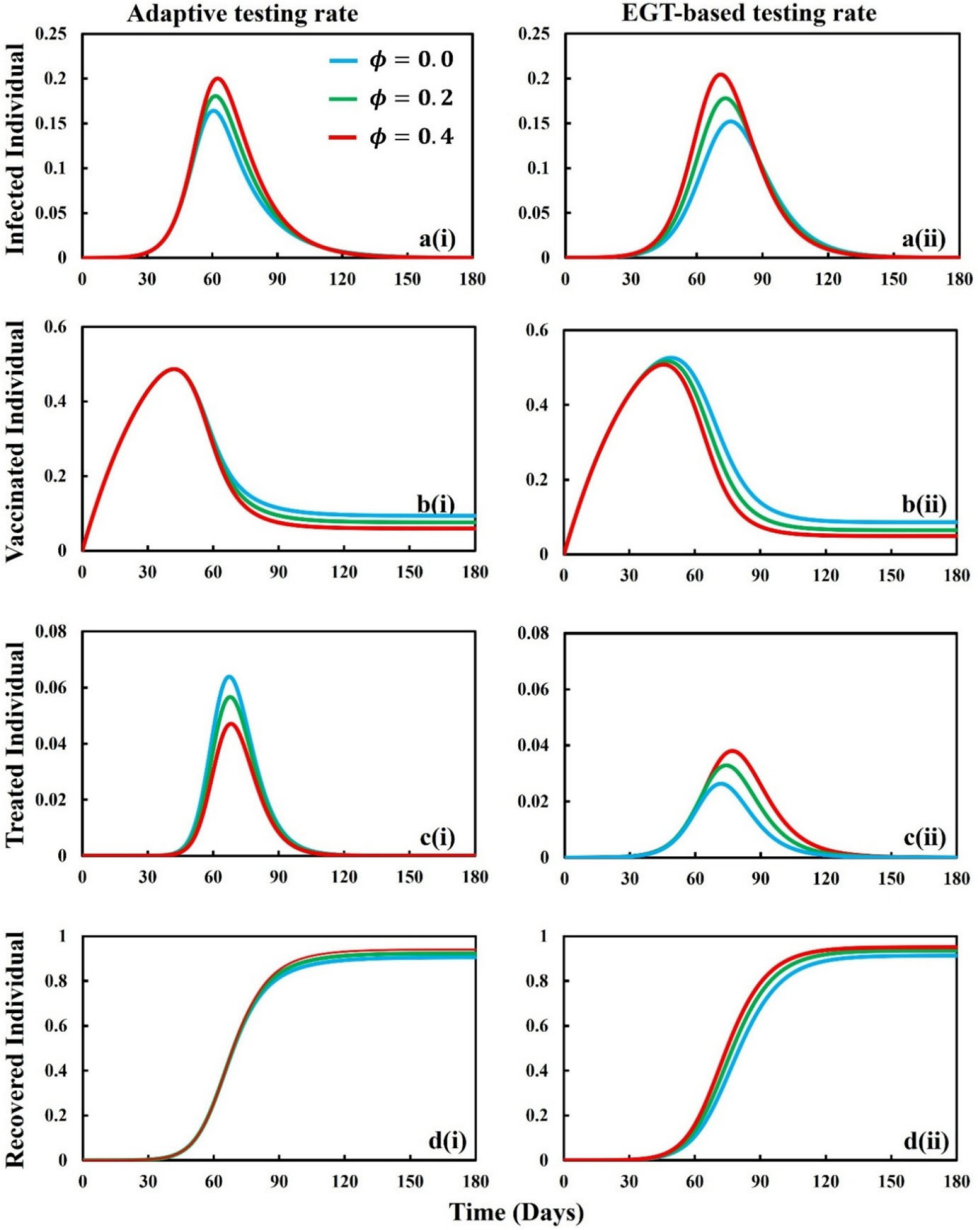
The effect of false negative results $(\phi =\mathrm{0.0,0.2,0.4})$ on the infected, vaccinated, treated, and recovered individuals through adaptive (panel i*-) and EGT-based (panel ii*-) testing rates. The parameter settings are $\beta =0.8333,\gamma =0.1,\alpha =0.2,{B}_{V}=0.2,a=0.2,\tau =0.9,\sigma =0.2,\omega =0.2,{m}_{x}={m}_{y}=0.1,{C}_{V}=0.5,\eta =0.5,{C}_{y}=0.1.$ For adaptive testing case, $k=0.1,\lambda =0.1,$ and EGT case, ${y}_{M}=0.2,\delta =0.1$ (Color figure online)

In an epidemic or pandemic, false-negative results considerably affect disease dynamics by compromising the efficacy of both adaptive and EGT-based testing methodologies (Fig. 9). In adaptive testing, a significant incidence of false negatives leads to underreporting of true infections, resulting in delayed or inadequate responses. This, in turn, perpetuates undiagnosed instances, undermining the efficacy of early detection and treatment strategies. In EGT-based testing, false negatives erode trust in the testing system. If people perceive that testing does not effectively identify infected individuals, they may forgo testing altogether, thereby reducing detection rates. Moreover, individuals with undiagnosed conditions may underestimate their risk, leading to a diminished perception of the need for immunization and reduced vaccine uptake. Consequently, false negatives facilitate unchecked illness proliferation by diminishing the detection and treatment of infected individuals, while undermining the behavioral feedback mechanisms essential to optimal decision-making in EGT models, thereby intensifying the progression of the epidemic.

**Fig. 10:**
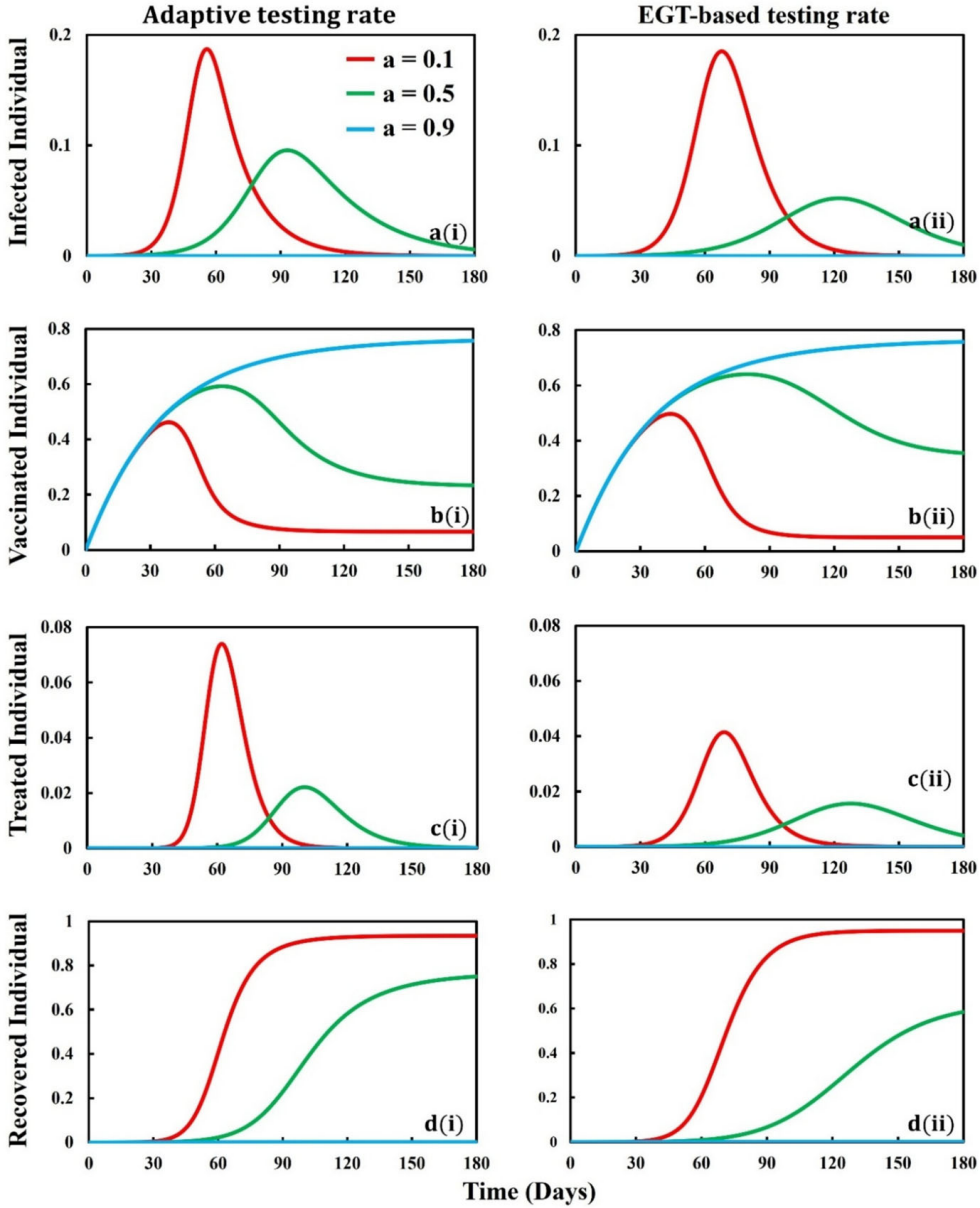
The effect of awareness $(a=\mathrm{0.1,0.5,0.9})$ on the infected, vaccinated, treated, and recovered individuals through adaptive (panel i*-) and EGT-based (panel ii*-) testing rates. The parameter settings are $\beta =0.8333,\gamma =0.1,\alpha =0.2,\phi =0.05,{B}_{V}=0.2,\tau =0.9,\sigma =0.2,\omega =0.2,{m}_{x}={m}_{y}=0.1,{C}_{V}=0.5,\eta =0.5,{C}_{y}=0.1.$ For adaptive testing case, $k=0.1,\lambda =0.1,$ and EGT case, ${y}_{M}=0.2,\delta =0.1$ (Color figure online)

Self-awareness significantly influences disease dynamics, particularly when testing behaviors are informed by strategies grounded in adaptive and evolutionary game theory (EGT). In adaptive testing frameworks, heightened individual awareness improves adherence to public health norms and promotes prompt testing, particularly in reaction to observable symptoms or documented outbreaks. This reactivity facilitates prompt identification, isolation, and treatment of affected individuals, thereby reducing overall transmission. In EGT-based testing, personal knowledge directly affects the perceived benefits of undergoing testing. Increased knowledge enhances the perceived risk of infection and the benefits of early identification, making people more likely to pursue testing, even without significant external incentives. This increased involvement, fueled by informed decision-making, facilitates faster case identification, bolsters contact tracing initiatives, and reduces the spread of infections. Ultimately, personal awareness enhances the efficacy of both testing procedures by promoting proactive behavior and strengthening community-level responses to disease outbreaks. According to the outcomes in Fig. 10, when the personal awareness rate is high ($a=0.9$), the disease is almost eradicated from society, and a significant number of people take the vaccine, as it is one of the most effective pharmaceutical interventions. Furthermore, fewer people need treatment because the infection rate is low; the same consequences apply to recovered cases.

**Fig. 11:**
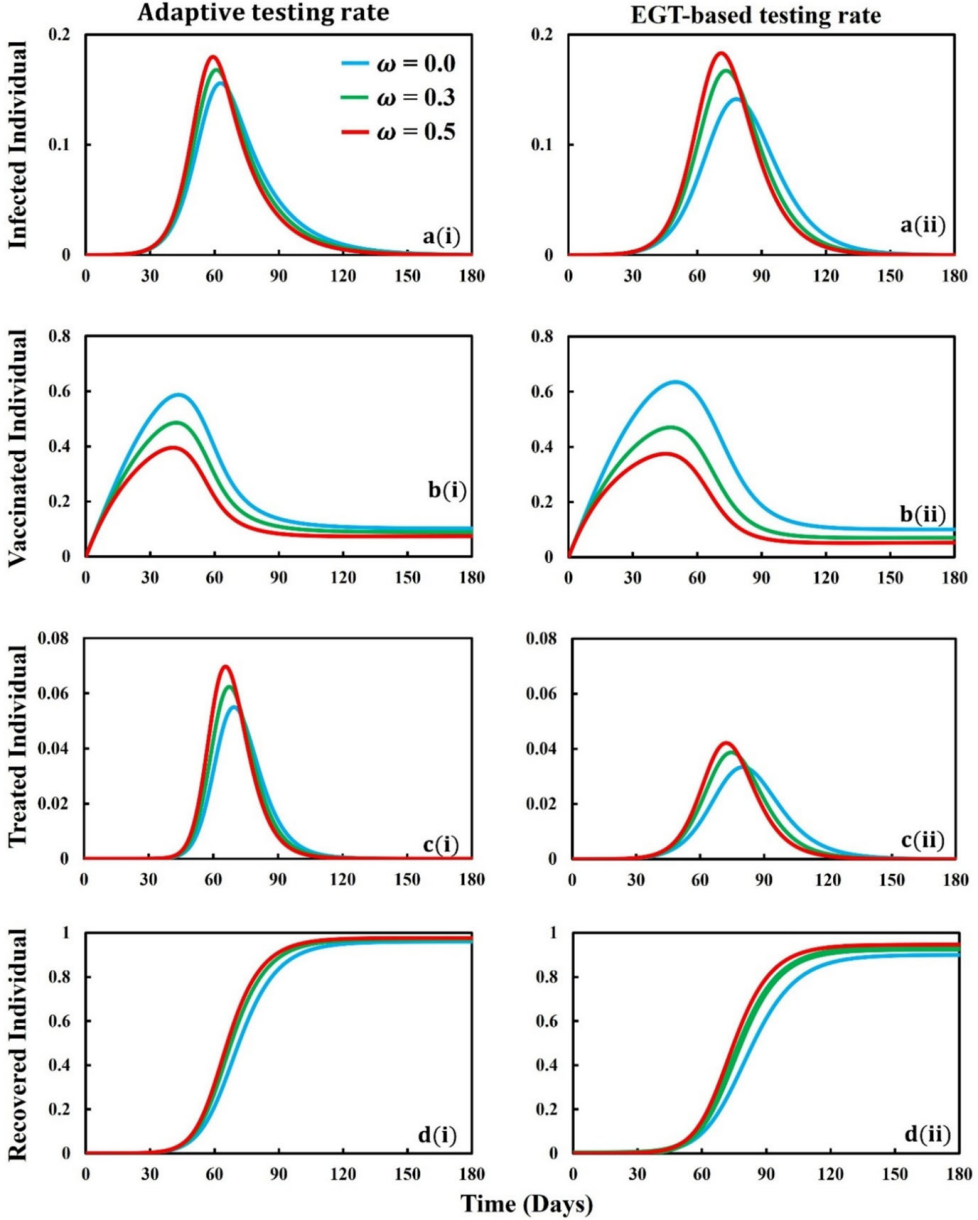
The effect of vaccination hesitancy $(\omega =\mathrm{0.0,0.3,0.5})$ on the infected, vaccinated, treated, and recovered individuals through adaptive (i*-) and EGT-based (ii*-) testing rates. The parameter settings are $\beta =0.8333,\gamma =0.1,\alpha =0.2,\phi =0.05,a=0.2,\tau =0.9,\sigma =0.2,{B}_{V}=0.2,{m}_{x}={m}_{y}=0.1,{C}_{V}=0.5,\eta =0.5,{C}_{y}=0.1.$ For adaptive testing case, $k=0.1,\lambda =0.1,$ and EGT case, ${y}_{M}=0.2,\delta =0.1$ (Color figure online)

Vaccine hesitancy or reluctance profoundly influences disease dynamics, as depicted in Fig. 11. In communities with significant vaccine reluctance, the overall immunization rate remains low, thereby increasing the vulnerable population and fostering conditions conducive to more extensive transmission (Fig. 11, red curve). In adaptive testing, the system can partially mitigate the problem by amplifying testing efforts as infections escalate, leading to earlier diagnosis and treatment. Nonetheless, in the absence of adequate vaccine coverage, the fundamental reason for transmission remains unaddressed, and outbreaks will continue despite extensive testing. Conversely, EGT-based testing, which relies on human decision-making influenced by social learning and perceived risk, will also be hindered by vaccination reluctance. Individuals who hesitate will also forgo testing if they assess the risks or inconveniences as outweighing the benefits, thereby diminishing the efficacy of early intervention. This hesitance undermines the feedback mechanism that often drives people to test and get vaccinated in response to heightened disease prevalence. Vaccine hesitancy ultimately reduces the synergistic impact of testing and vaccination, thereby prolonging the epidemic or pandemic and exacerbating strain on healthcare systems, despite the implementation of well-structured, adaptive, or EGT-based testing procedures.

**Fig. 12:**
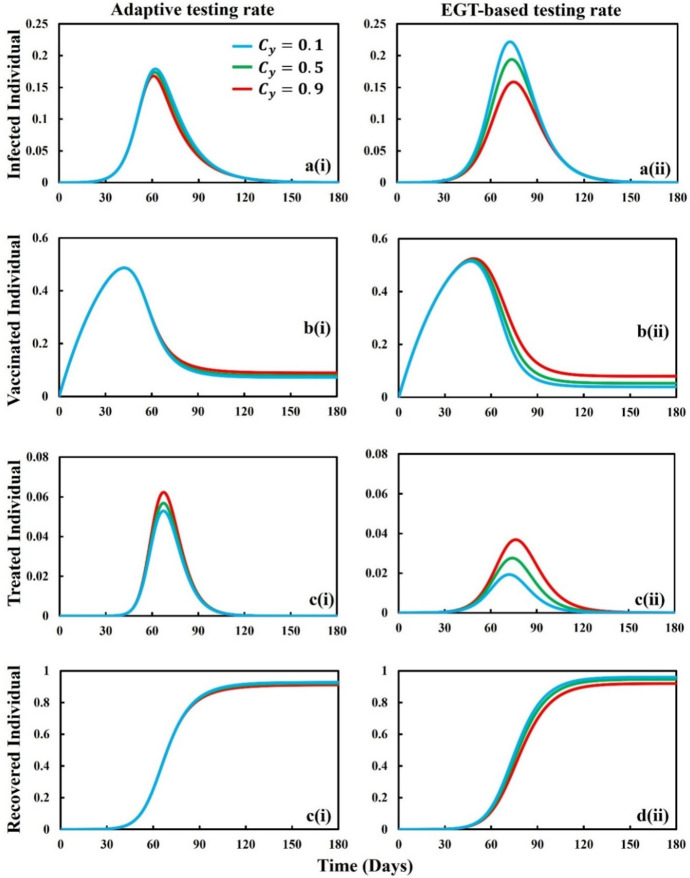
The effect of testing cost $({C}_{y}=\mathrm{0.1,0.5},0.9)$ on the infected, vaccinated, treated, and recovered individuals through adaptive (i*-) and EGT-based (ii*-) testing rates. The parameters setting are $\beta =0.8333,\gamma =0.1,\alpha =0.2,\phi =0.05,a=0.2,\tau =0.9,\sigma =0.2,{B}_{V}=0.2,{m}_{x}={m}_{y}=0.1,{C}_{V}=0.5,\eta =0.5,\omega =0.2.$ For adaptive testing case, $k=0.1,\lambda =0.1,$ and EGT case, ${y}_{M}=0.2,\delta =0.1$ (Color figure online)

Figure 12 represents the effect of testing costs on proposed models of disease dynamics. There is no denying that testing costs significantly influence disease dynamics, particularly when testing behaviors are driven by adaptive strategies or by EGT-based decision-making. In adaptive testing, external factors such as public health policy or monitoring systems often influence the assessment and exhibit relative stability irrespective of individual cost factors. Nevertheless, higher cost exerts pressure on public health resources. It reduces the frequency or scope of adaptive testing, thereby delaying the identification and isolation of infected individuals and their transfer for treatment, which ultimately leads to increased transmission. Conversely, with EGT-based testing, people evaluate the expense of testing in terms of the anticipated benefit of knowing their infection status. As testing costs rise, fewer people choose to test, particularly when the perceived risk of infection is low. It is evident that when testing costs are high, many exposed and infected individuals do not participate in testing and remain undetected in the population. They transmit the disease to society, which is not an impeccable outcome for any society, and they act like a social disease bomb. In that case, policymakers cannot control disease transmission in society through a straightforward mechanism. Vaccinated individuals may opt not to undergo testing due to perceived reduced risk, mainly when testing costs are high, which could result in missed breakthrough infections. Individuals who have received treatment need follow-up testing, and high costs can impede effective post-treatment monitoring. Recovered individuals also require tests to verify complete recovery or reinfection, and high testing costs could restrict their access to these services. This results in underreporting, delayed treatment, and an increased transmission probability, thereby intensifying the epidemic. Consequently, elevated testing costs in both frameworks hinder early diagnosis and containment, with the effect more pronounced in EGT-based testing, where individual choice prevails. Finally, minimizing testing costs or providing incentives promotes broader participation, enhances early diagnosis, and facilitates more efficient disease control in adaptive, strategic testing contexts.

**Fig. 13:**
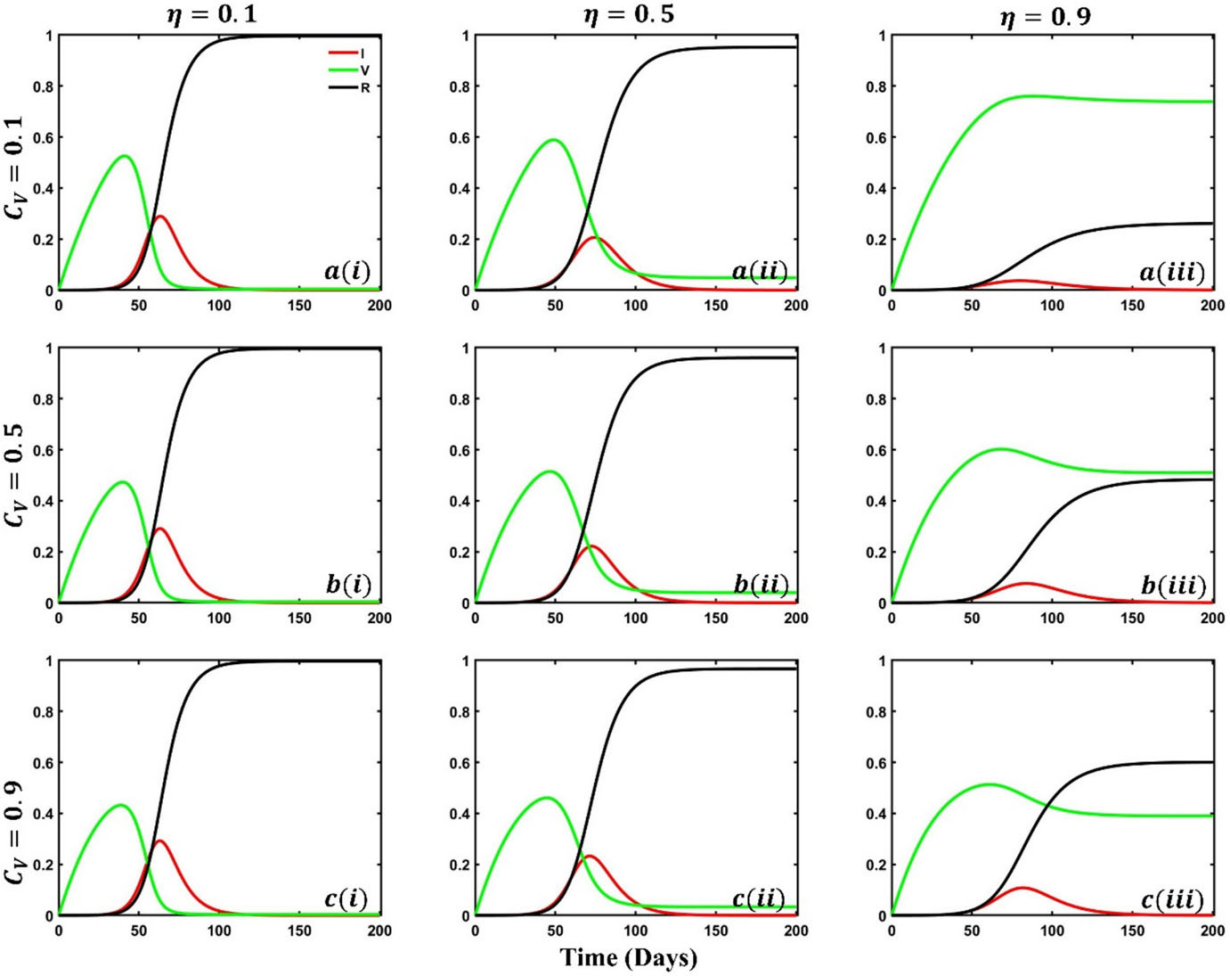
The effect of vaccination cost $({C}_{V}=\mathrm{0.1,0.5,0.9})$ and efficacy $(\eta =\mathrm{0.1,0.5,0.9})$ on the infected, vaccinated, and recovered individuals through EGT-based testing and vaccination rates. Other parameters setting are as follows: $\beta =0.8333,\gamma =0.1,\alpha =0.2,\phi =0.05,a=0.2,\tau =0.9,\sigma =0.2,{B}_{V}=0.2,{m}_{x}={m}_{y}=0.1,{C}_{V}=0.5,\eta =0.5,\delta =0.1,\omega =0.2,{y}_{M}=0.2$ (Color figure online)

Figure 13 displays the time-series plots illustrating the dynamics of the vaccinated $(V)$, infected $(I={U}_{I}+{D}_{I})$, and recovered $(R)$ populations, under different conditions of vaccination cost $({C}_{V}=0.1, 0.5, 0.9)$ and efficacy of vaccine $(\eta =0.1, 0.5, 0.9)$ within evolutionary game theory (EGT)-based testing and vaccination game. Here, $x$-axis represents time (in days), whilst the $y$-axis signifies the fraction of the population inside each class. Each row represents increasing immunization costs, while each column denotes increasing effectiveness. In all subfigures of Fig. 13, the green curve represents the infected population, the red curve the vaccinated population, and the black curve the recovered population. When both effectiveness and cost are minimal (top-left panel $a(i)$), infections surge rapidly and predominate the epidemic trajectory, whereas vaccination is limited and recovery occurs mostly through spontaneous disease progression. As vaccine effectiveness improves (moving to the right), the infection peak decreases, recovery becomes more gradual, and vaccination becomes more efficient in flattening the epidemic curve. In addition, in the rightmost column $(\eta =0.9)$, vaccination noticeably reduces the transmission of infections in the early stages, particularly when costs are minimal $({C}_{V}=0.1)$, resulting in a substantial and enduring vaccinated population with a postponed and diminished infection peak. In contrast, higher vaccination costs (${C}_{V}=0.9$, bottom row) limit vaccination despite its significant effectiveness, leading to a larger residual infected population and a more gradual increase in recovered persons. In summary, Fig. 13 shows that a combination of high vaccination effectiveness and low cost is the most effective approach for rapidly reducing infection rates and enhancing population immunity. Simultaneously, elevated vaccination costs impede adoption despite the vaccine’s efficacy, highlighting the significance of economic accessibility in epidemic control measures influenced by human choices and adaptive behavior.

**Fig. 14:**
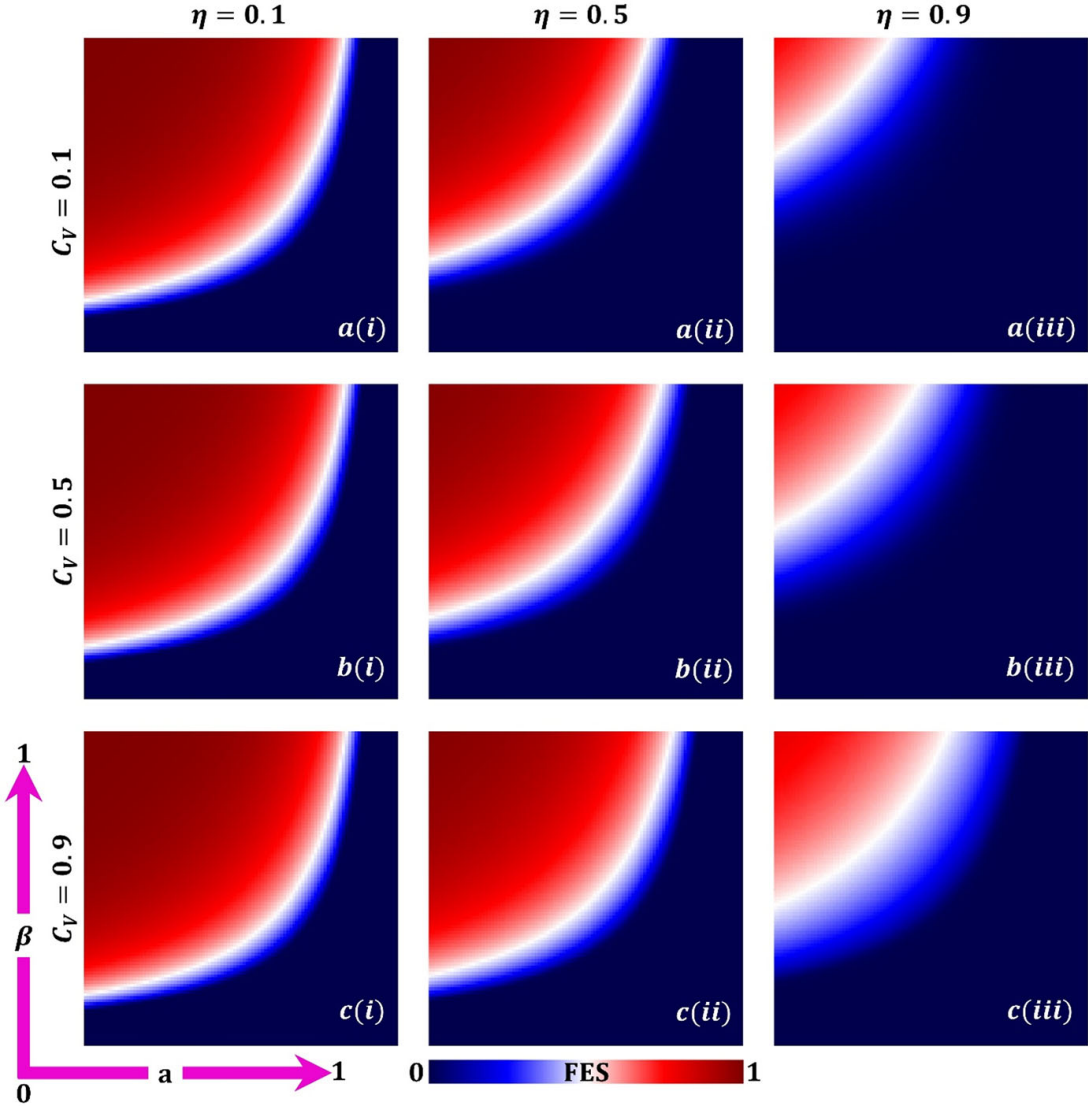
2D graphical representation of the final epidemic size (FES) of disease dynamics based on the vaccination cost $({C}_{V}=\mathrm{0.1,0.5,0.9})$ and efficacy $(\eta =\mathrm{0.1,0.5,0.9})$ with adaptive testing and EGT-based vaccination rates, where the remaining parameter values are as follows: $\alpha =0.2,\gamma =0.1,\phi =0.05,\tau =0.9,\sigma =0.3,\delta =0.1,{m}_{x}=0.1, {B}_{V}=0.2,\omega =0.05,\lambda =0.3,k=0.1,{C}_{y}=0.1$. $x$-axis designates personal awareness rate $(a),$ and the $y$-axis specifies the disease transmission rate $(\beta )$ (Color figure online)

**Fig. 15:**
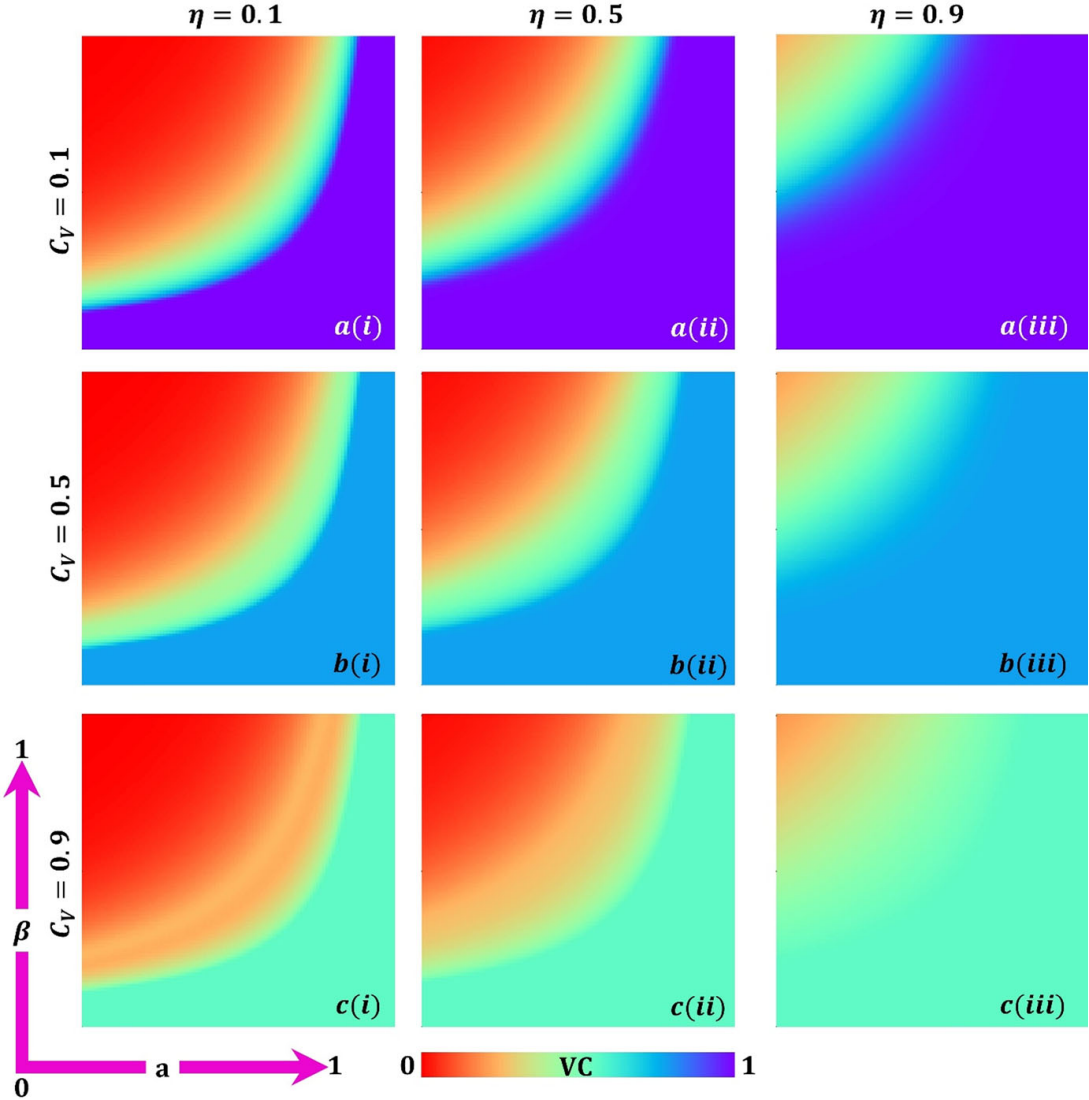
2D graphical representation of vaccination coverage (VC) of disease dynamics based on the vaccination cost $({C}_{V}=\mathrm{0.1,0.5,0.9})$ and efficacy $(\eta =\mathrm{0.1,0.5},0.9)$ with adaptive testing and EGT-based vaccination rates, where the remaining parameter values are as follows: $\alpha =0.2,\gamma =0.1,\phi =0.05,\tau =0.9,\sigma =0.3,\delta =0.1,{m}_{x}=0.1, {B}_{V}=0.2,\omega =0.05,\lambda =0.3,k=0.1,{C}_{y}=0.1$. $x$-axis designates personal awareness rate $(a),$ and the $y$-axis specifies the disease transmission rate $(\beta )$ (Color figure online)

**Fig. 16:**
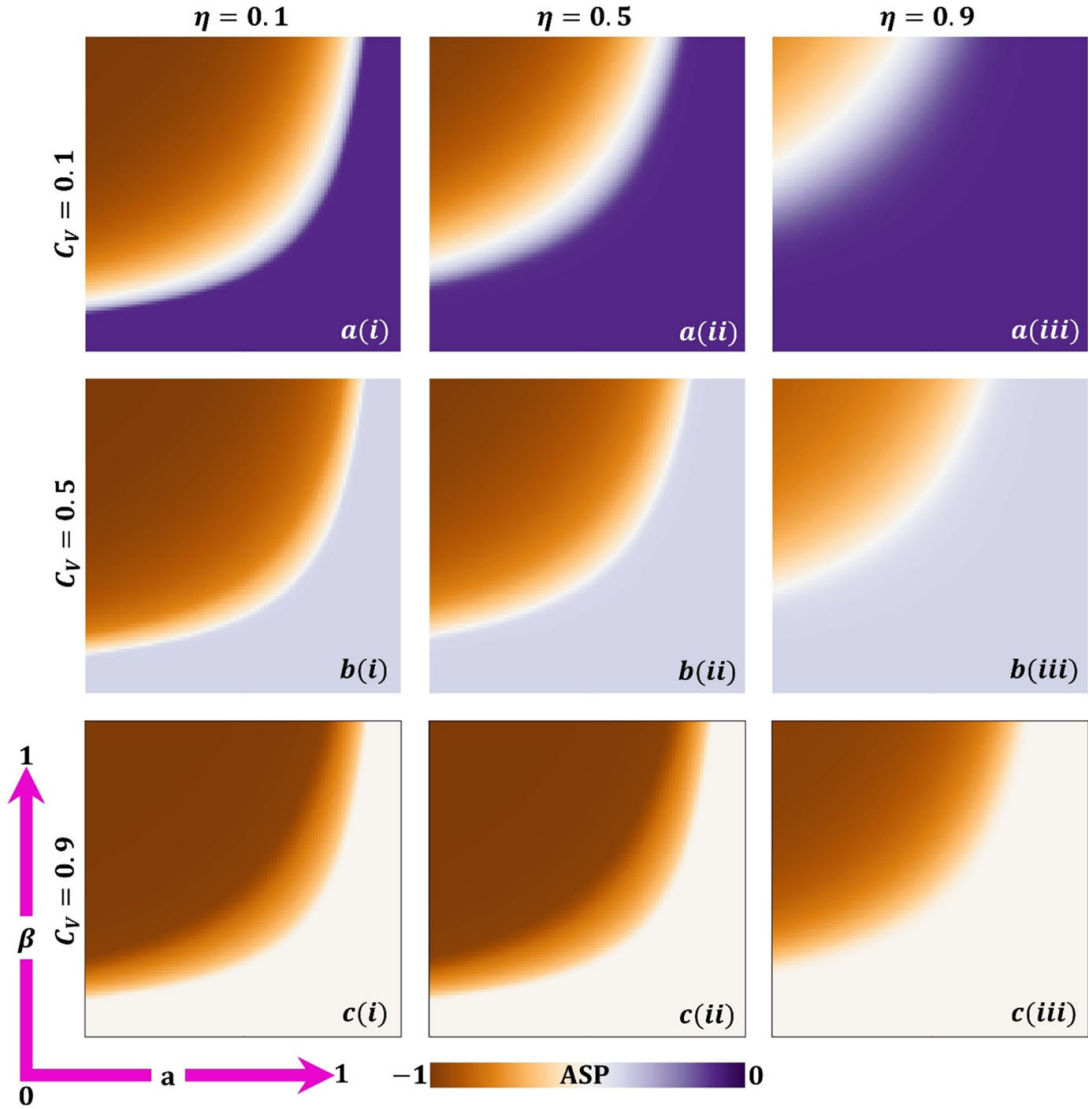
2D graphical representation of average social payoff (ASP) of disease dynamics based on the vaccination cost $({C}_{V}=\mathrm{0.1,0.5,0.9})$ and efficacy $(\eta =\mathrm{0.1,0.5,0.9})$ with adaptive testing and EGT-based vaccination rates, where the remaining parameter values are as follows: $\alpha =0.2,\gamma =0.1,\phi =0.05,\tau =0.9,\sigma =0.3,\delta =0.1,{m}_{x}=0.1, {B}_{V}=0.2,\omega =0.05,\lambda =0.3,k=0.1,{C}_{y}=0.1$. $x$-axis designates personal awareness rate $(a),$ and the $y$-axis specifies the disease transmission rate $(\beta )$ (Color figure online)

Figures 14, 15, 16 encompass the 2D heatmap panels that represent the effects of vaccination cost $({C}_{V}=0.1, 0.5, 0.9)$ and vaccine efficacy $(\eta =0.1, 0.5, 0.9)$ on the final epidemic size (FES) (Fig. 14), vaccination coverage (VC) (Fig. 15), and average social payoff (ASP) (Fig. 16) (Ouardani et al. 2026; Ullah and Wang 2025) within a framework that integrates adaptive testing and an EGT-based vaccination game. The 2D heatmaps are delineated with the awareness level $a$ on the $x$-axis and the disease transmission rate $\beta$ on the $y$-axis, using factors representing epidemiological and behavioral settings. In the FES of Fig. 14, the red regions indicate a larger epidemic scale, while the blue represents outbreak suppression, specifically a higher disease-free equilibrium scenario. The heatmaps indicate that FES significantly diminishes with increased awareness and reduced transmission rates. Enhancing vaccine effectiveness $(\eta )$ significantly lowers the red-blue border, indicating that epidemics are controlled even at elevated transmission rates when $\eta$ is robust. Nonetheless, escalating vaccination costs $({C}_{V})$ mitigate this impact by diminishing the motivation to vaccinate, especially when $\eta$ is low. Again, blue-to-purple indicates high coverage in VC heatmaps (Fig. 15), which increases with greater knowledge and effectiveness, though it declines significantly with higher expense. High efficacy still yields moderate-to-high VC despite intermediate costs, particularly when awareness is sufficiently high, underscoring the nonlinear relationship between economic stress and perceived health gain in shaping behavioral responses. Furthermore, Fig. 16 displays the average social payoff (ASP), where orange indicates a low reward (− 1), and purple indicates a greater reward (0), which increases with increased awareness and reduced transmission, aligning with the trends observed in FES and VC in Figs. 14 and 15. When both ${C}_{V}$ is minimal and $\eta$ is maximal, ASP attains its peak, indicating both personal awareness and reduced societal cost. Therefore, statistics underscore the essential relationships among cost, effectiveness, and awareness in shaping the outcomes of epidemics. When combined with behavior-driven vaccination tactics, adaptive testing demonstrates that enhancing public health communication (awareness) and ensuring economic accessibility of vaccines substantially improve population-level protection and social benefits.

**Fig. 17:**
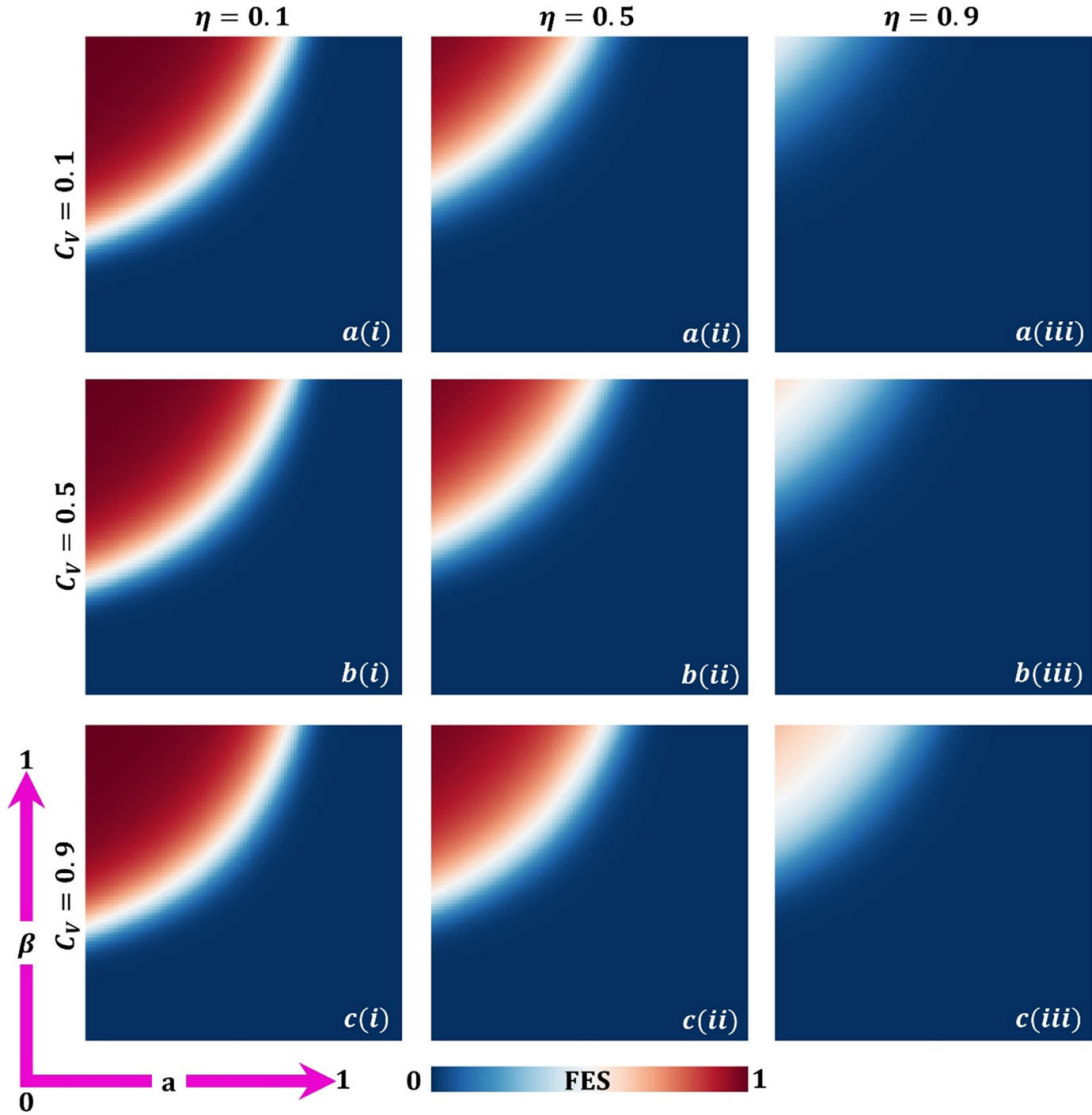
2D graphical representation of the final epidemic size (FES) of disease dynamics based on vaccination cost $({C}_{V}=\mathrm{0.1,0.5,0.9})$ and efficacy $(\eta =\mathrm{0.1,0.5,0.9})$ with EGT-based testing and vaccination rates, where the remaining parameter settings are as follows: $\alpha =0.2,\gamma =0.1,\phi =0.05,\tau =0.9,\sigma =0.2,\delta =0.1, {{m}_{x}=0.1,\omega =0.2,B}_{V}=0.2,{m}_{y}=0.1,{C}_{y}=0.1,{y}_{M}=0.2$. $x$-axis designates awareness level $(a),$ and the $y$-axis specifies the disease transmission rate $(\beta )$ (Color figure online)

**Fig. 18:**
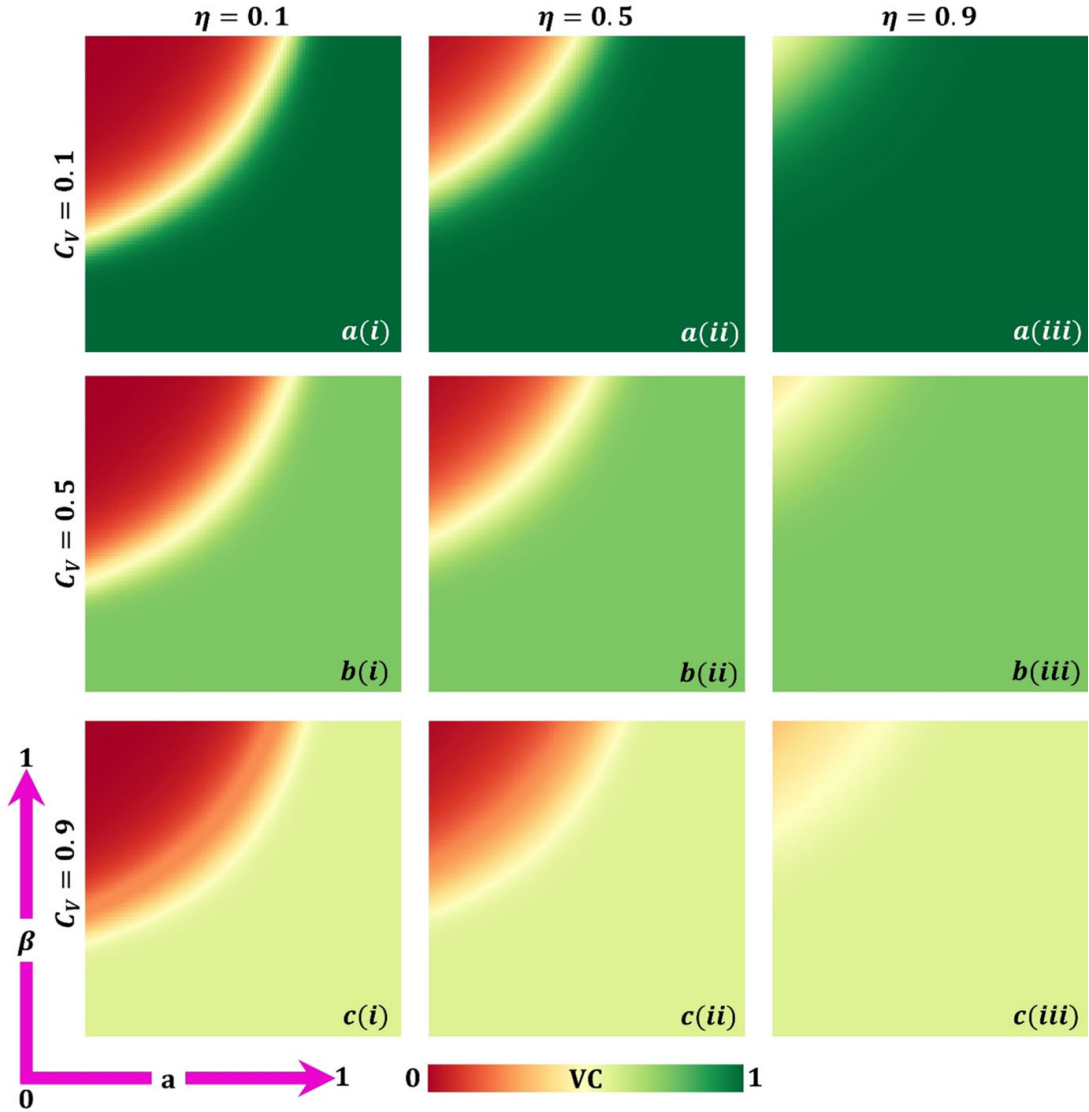
2D graphical representation of vaccination coverage (VC) of disease dynamics based on vaccination cost $({C}_{V}=\mathrm{0.1,0.5,0.9})$ and efficacy $(\eta =\mathrm{0.1,0.5,0.9})$ with EGT-based testing and vaccination rates, where the remaining parameter settings are as follows: $\alpha =0.2,\gamma =0.1,\phi =0.05,\tau =0.9,\sigma =0.2,\delta =0.1, {{m}_{x}=0.1,\omega =0.2,B}_{V}=0.2,{m}_{y}=0.1,{C}_{y}=0.1,{y}_{M}=0.2$. $x$-axis designates awareness level $\left(a\right),$ and the $y$-axis specifies the disease transmission rate $(\beta )$ (Color figure online)

**Fig. 19:**
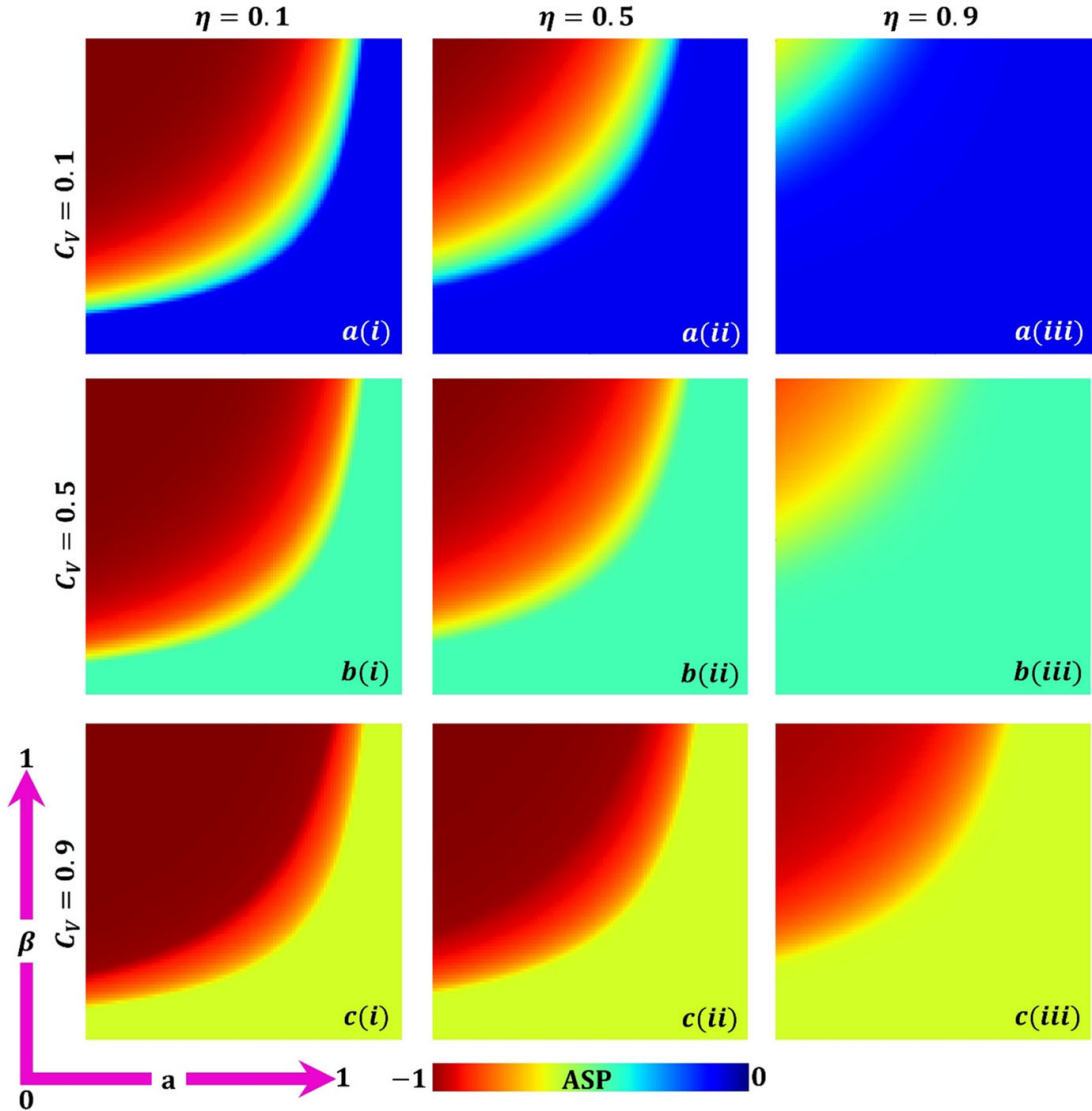
2D graphical representation of average social payoff (ASP) of disease dynamics based on vaccination cost $({C}_{V}=\mathrm{0.1,0.5,0.9})$ and efficacy $(\eta =\mathrm{0.1,0.5,0.9})$ with EGT-based testing and vaccination rates, where the remaining parameter values are as follows: $\alpha =0.2,\gamma =0.1,\phi =0.05,\tau =0.9,\sigma =0.2,\delta =0.1, {{m}_{x}=0.1,\omega =0.2,B}_{V}=0.2,{m}_{y}=0.1,{C}_{y}=0.1,{y}_{M}=0.2$. $x$-axis designates awareness level $(a),$ and the $y$-axis specifies the disease transmission rate $(\beta )$ (Color figure online)

Figures 17, 18, 19 underscore the importance of vaccination effectiveness, cost, and public awareness in controlling outbreaks, enhancing social benefit, and fostering behavioral compliance within game-theoretic (testing and vaccination) epidemic dynamics. These figures illustrate the effects of the cost of vaccination $({C}_{V}=0.1, 0.5, 0.9)$ and vaccine efficacy $(\eta = 0.1, 0.5, 0.9)$ on FES, VC, and ASP, within a framework based on EGT, where the $x$-axis designates awareness level $(a)$ and the $y$-axis specifies the transmission rate $(\beta )$. In the case of the FES (Fig. 17), blue areas specify minimal epidemic sizes (effective control), whereas red areas denote substantial outbreaks. The findings show that augmenting $a$ or diminishing $\beta$ decreases FES, whereas elevated vaccination effectiveness improves control, an expected outcome. As ${C}_{V}$ intensifies (from top to bottom), the blue zone diminishes, indicating that elevated vaccination costs deter uptake and lead to larger outbreaks. Similarly, the VC panel (Fig. 18) corroborates these findings, with green indicating good vaccination uptake and red indicating inadequate coverage. The effectiveness of vaccines is paramount: even at elevated costs, an increase in $\eta$ enhances vaccination acceptance, particularly when awareness levels are high. Lower $\eta$ or higher ${C}_{V}$ significantly diminishes VC unless $a$ is high and $\beta$ is minimal. Finally, Fig. 19 assesses the overall societal benefit from $-1$ (red) to 0 (blue). A higher ASP (approaching blue) is observed in regions with low vaccination costs, high effectiveness, and higher VC, as well as diminished FES. As ${C}_{V}$ heightens or $\eta$ diminishes, ASP decreases, particularly at high disease transmission rates, highlighting the economic and health repercussions of an inadequate vaccine response, rather than achieving a better outcome for any society.

**Fig. 20:**
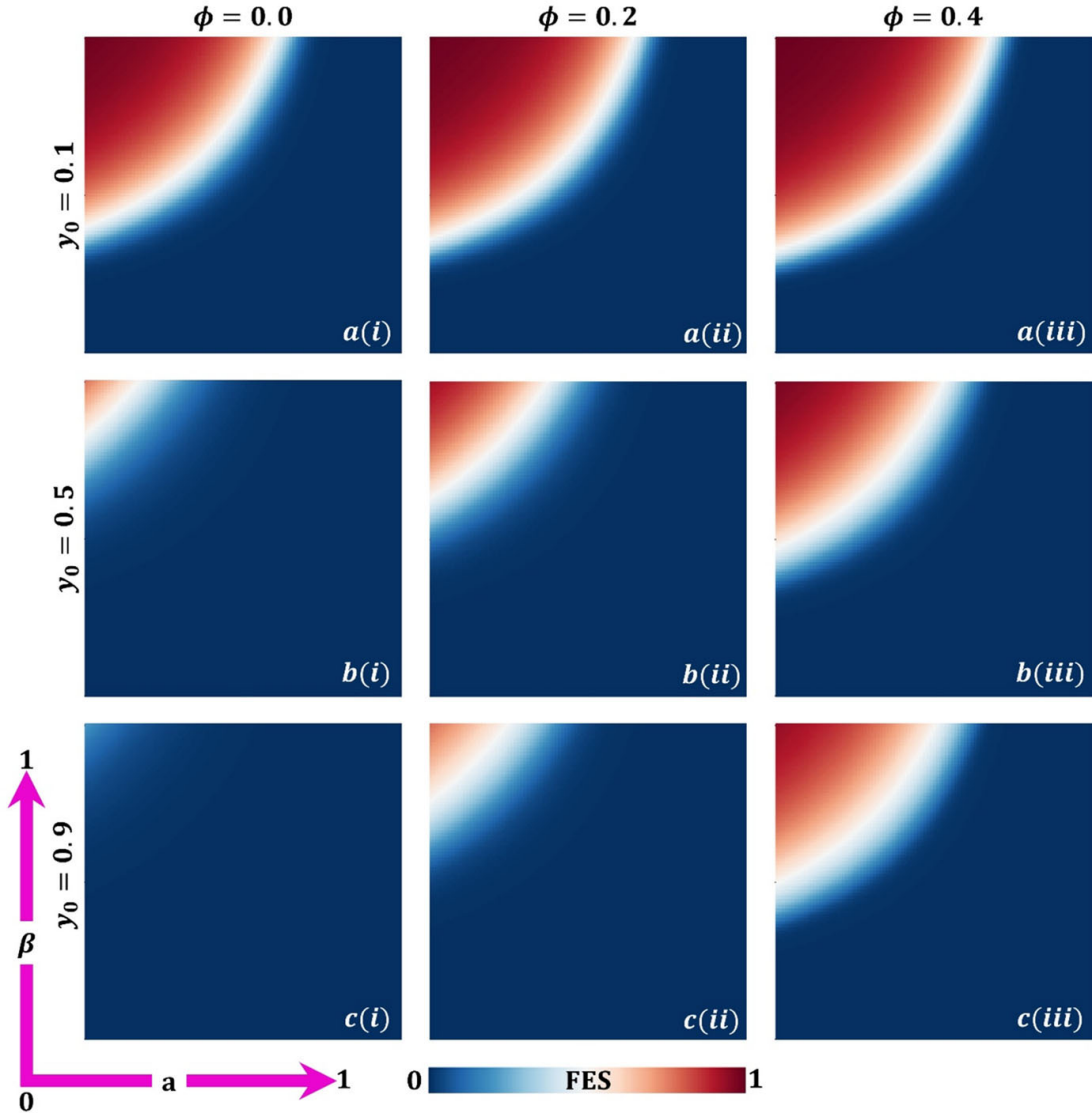
2D graphical representation of final epidemic size (FES) of disease dynamics based on testing rate $({y}_{0}=\mathrm{0.1,0.5,0.9})$ and false negative results rate $(\phi =\mathrm{0.0,0.2,0.4})$ with EGT-based testing and vaccination rates, where the remaining parameter values are as follows: $\alpha =0.2,\gamma =0.1,\eta =0.5,\tau =0.9,\sigma =0.2,\delta =0.1,{m}_{x}=0.1, {C}_{V}=0.5,{B}_{V}=0.2,{\omega =0.2,m}_{y}=0.1,{C}_{y}=0.1,{y}_{M}=0.2$. $x$-axis designates awareness level $(a),$ and the $y$-axis specifies the disease transmission rate $(\beta )$ (Color figure online)

**Fig. 21:**
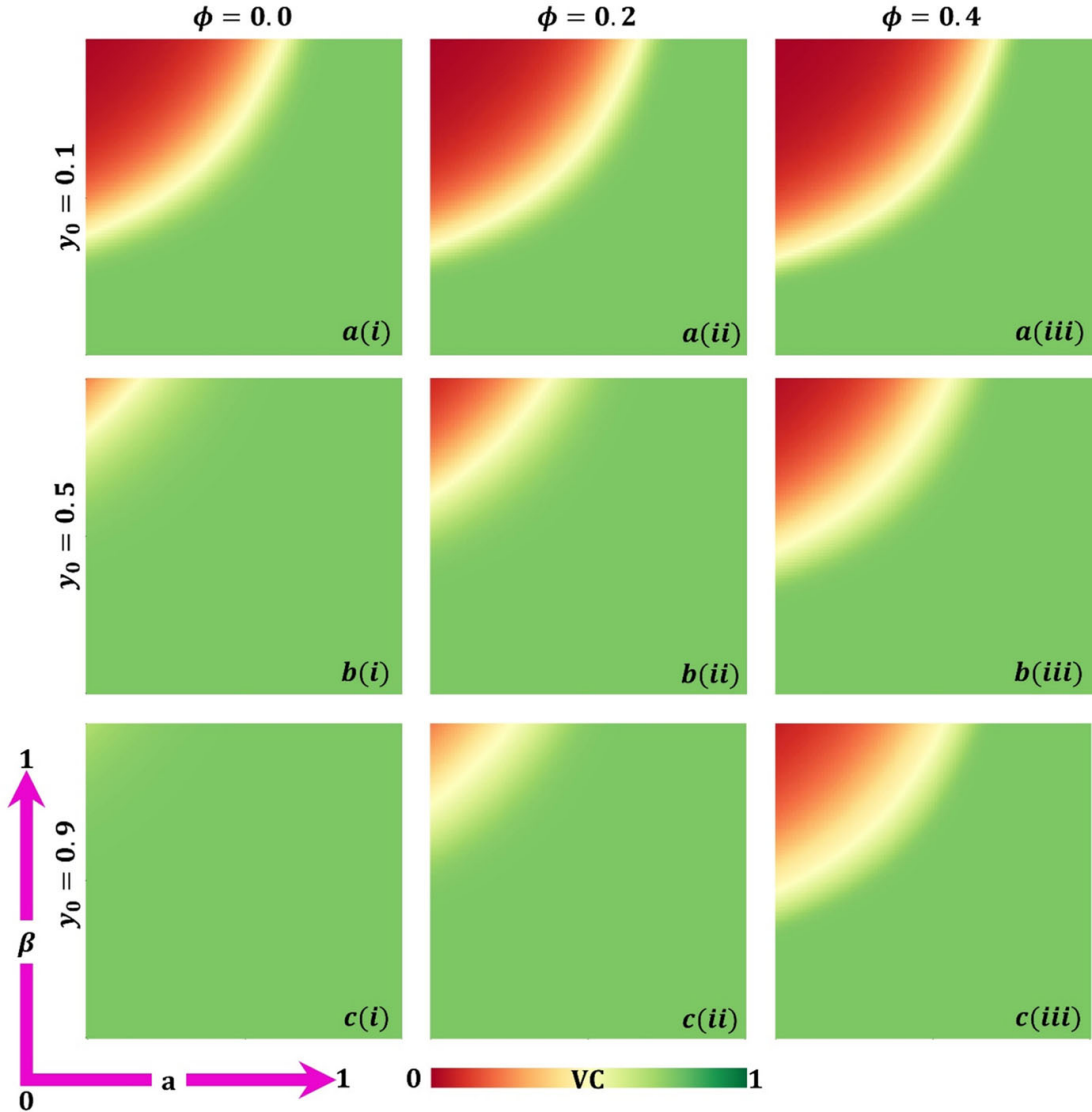
2D graphical representation of vaccination coverage (VC) of disease dynamics based on testing rate $({y}_{0}=\mathrm{0.1,0.5,0.9})$ and false negative results rate $(\phi =\mathrm{0.0,0.2,0.4})$ with EGT-based testing and vaccination rates, where the remaining parameter values are as follows: $\alpha =0.2,\gamma =0.1,\eta =0.5,\tau =0.9,\sigma =0.2,\delta =0.1,{m}_{x}=0.1, {C}_{V}=0.5,{B}_{V}=0.2,{\omega =0.2,m}_{y}=0.1,{C}_{y}=0.1,{y}_{M}=0.2$. $x$-axis designates awareness level $(a),$ and the $y$-axis specifies the disease transmission rate $(\beta )$ (Color figure online)

**Fig. 22:**
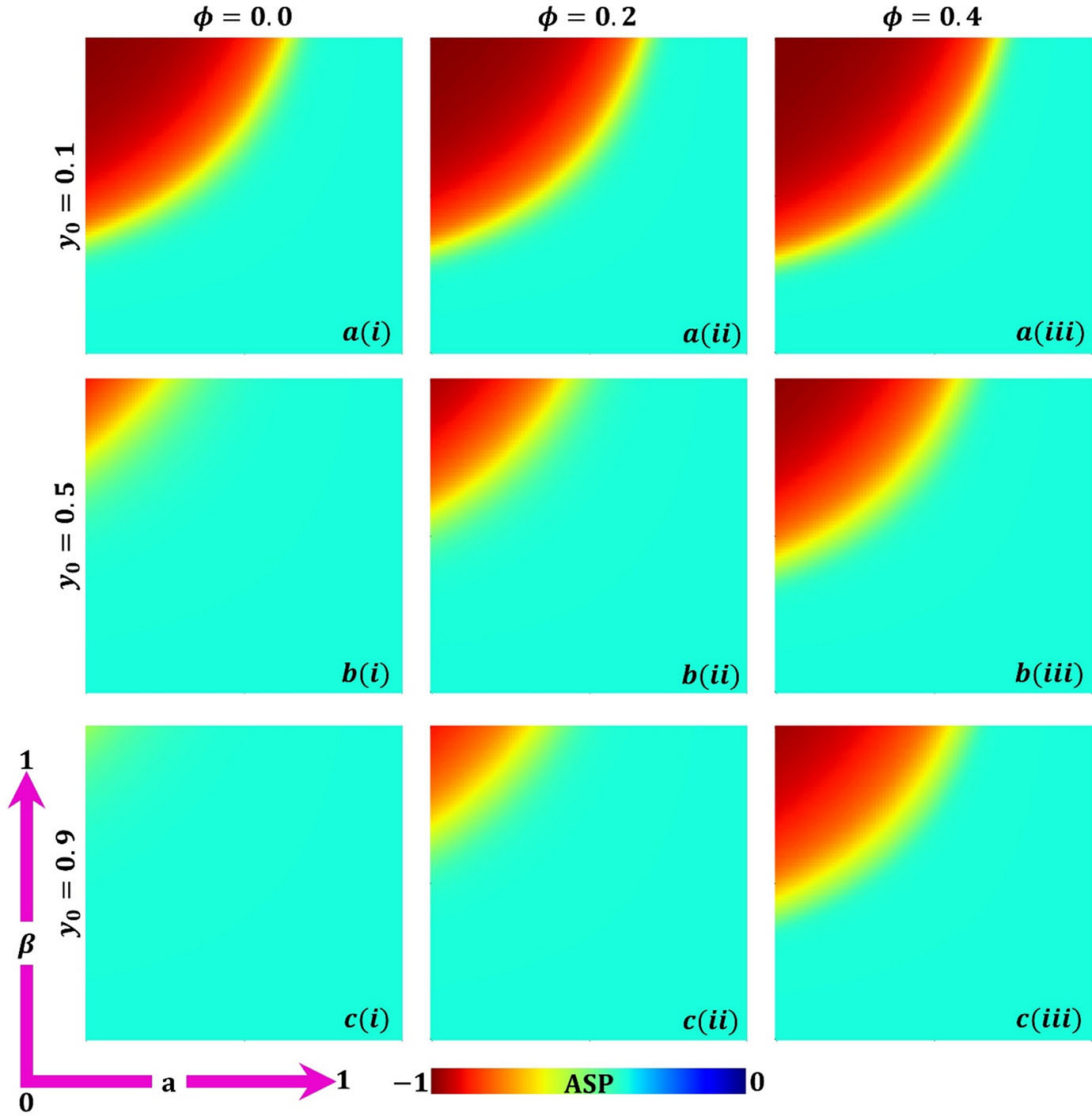
2D graphical representation of average social payoff (ASP) of disease dynamics based on testing rate $({y}_{0}=\mathrm{0.1,0.5,0.9})$ and false negative results rate $(\phi =\mathrm{0.0,0.2,0.4})$ with EGT-based testing and vaccination rates, where the remaining parameter values are as follows: $\alpha =0.2,\gamma =0.1,\eta =0.5,\tau =0.9,\sigma =0.2,\delta =0.1,{m}_{x}=0.1, {C}_{V}=0.5,{B}_{V}=0.2,{\omega =0.2,m}_{y}=0.1,{C}_{y}=0.1,{y}_{M}=0.2$. $x$-axis designates awareness level $(a),$ and the $y$-axis specifies the disease transmission rate $(\beta )$ (Color figure online)

Figures 20, 21, 22 illustrate the FES, VC, and ASP of disease dynamics (EGT-based testing and vaccination) as a function of the transmission rate ($\beta$, $x$-axis) and personal awareness or protection rate ($a$, $y$-axis), across various initial testing rates (${y}_{0}=0.1, 0.5, 0.9$) and testing reports false negative rates $(\phi =0.0, 0.2, 0.4)$. Each grid row denotes ascending values of the initial testing rate ${y}_{0}$, whilst each column indicates the intensifying diagnostic reports error ($\phi$). In Fig. 20, the color gradient transitions from blue (indicating low FES, or successful epidemic control) to red (indicating high FES, or widespread infection). When the testing rate is minimal (${y}_{0}=0.1$, top row), the epidemic's magnitude is acutely responsive to increases in β, particularly as false-negative rates rise, since undiagnosed cases facilitate transmission. As the initial testing rate escalates (${y}_{0}=0.5$ and ${y}_{0}= 0.9$), the blue zones become more noticeable, especially at intermediate levels of personal awareness or protection and disease transmission, indicating substantial epidemic suppression driven by early diagnosis and control. Despite significant testing uncertainty ($\phi =0.4$), a high testing rate (${y}_{0}=0.9$) sustains low FES, underscoring that regular testing is an effective strategy for epidemic control and can mitigate the limitations of suboptimal test precision. This result highlights the importance of comprehensive, proactive testing initiatives and public awareness campaigns in mitigating the severity of epidemics within the EGT framework. On the VC heatmaps in Fig. 21, green areas signify high vaccination uptake, while red regions indicate lower uptake. At low *y*_0_ (top row), vaccination coverage is acutely sensitive to both *∅* and *β*, with coverage declining precipitously in areas of elevated transmission and testing reports inaccuracies. As *y*₀ grows, the green zone extends, indicating that more early testing correlates with higher vaccine adoption, even with elevated ϕ. Increasing awareness rate $a$ and modest disease transmission $\beta$ further substantiate this impact. Finally, Fig. 22, the ASP heatmaps, exemplifying the aggregate population-level advantage of strategic activity, display payoff values from $-1$ (red) to 0 (blue). At low ${y}_{0},$ ASP is significantly reduced when $\phi$ is high and transmission is vigorous, owing to inadequate regulation and financial strain. Nonetheless, as ${y}_{0}$ increases, the ASP exhibits a marginal improvement over most of the domain, while it continues to be suboptimal (cyan to red colors), particularly at elevated values of $\phi$. Therefore, early and extensive testing is crucial for promoting vaccination participation and achieving better societal outcomes. Furthermore, lessening false negative results is essential for sustaining high vaccination uptake and favorable social outcomes amidst increased disease transmissibility in any society.

**Fig. 23:**
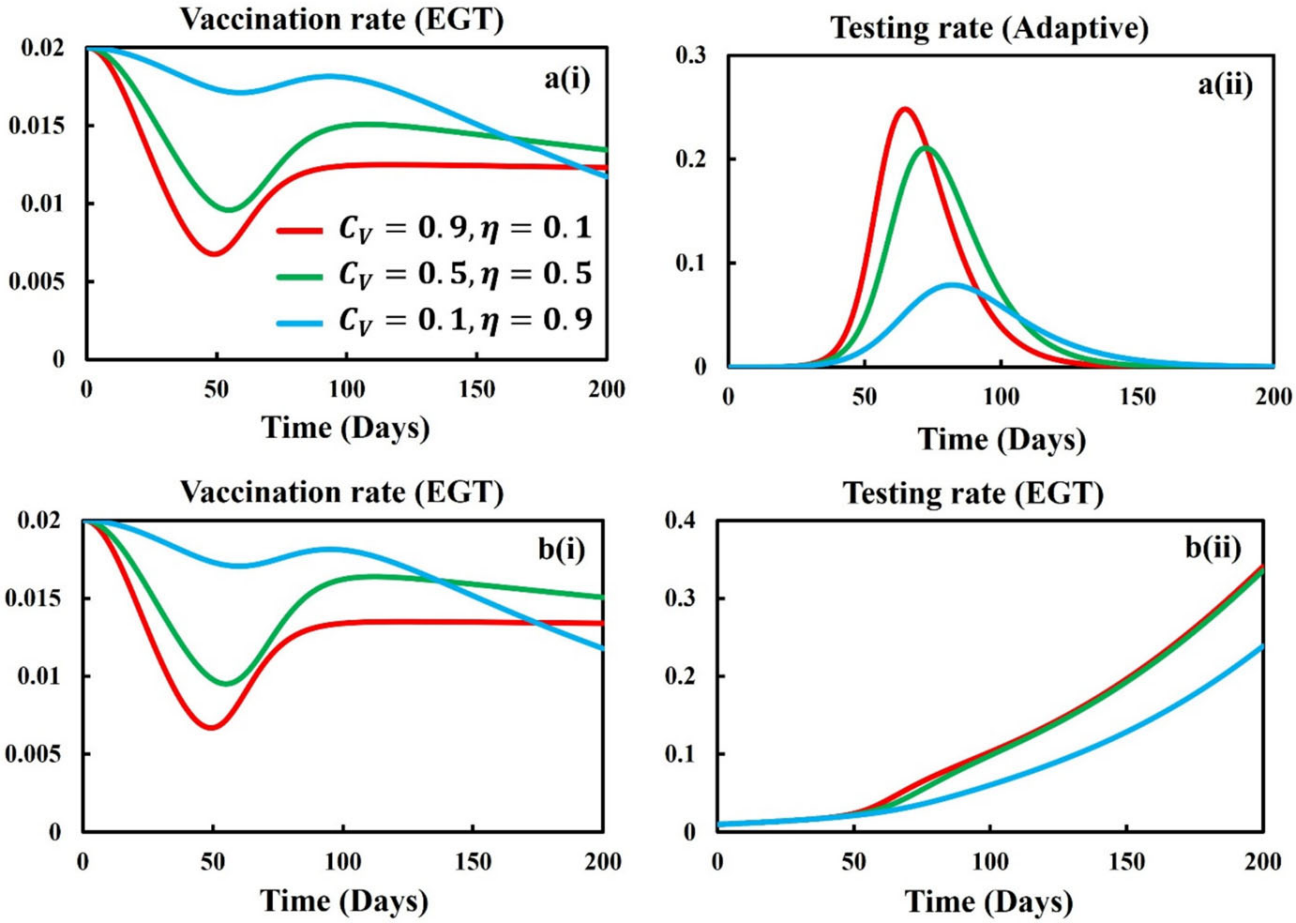
Effect of vaccination and testing rates for different values of vaccination cost and efficacy ($\left({C}_{V},\eta \right)=\left(\mathrm{0.9,0.1}\right),\left(\mathrm{0.5,0.5}\right),(\mathrm{0.1,0.9})$). The remaining parameter values are as follows: $\beta =0.8333,a=0.2,\alpha =0.2,\gamma =0.1,\phi =0.05,\tau =0.8,\sigma =0.2,\delta =0.15,{m}_{x}=0.1, {C}_{V}=0.5{,m}_{y}=0.1,{C}_{y}=0.1,k=0.15,\lambda =0.1$ (Color figure online)

Figure 23 illustrates how evolutionary game theory (EGT) governs vaccination and testing across scenarios of vaccination cost and effectiveness, with testing either determined adaptively or via EGT. Left panels show vaccination rate dynamics ($x(t)$) and right panels compare testing rate dynamics ($y(t)$) using adaptive testing (top) and EGT-based testing (bottom). Lower vaccination cost and higher vaccine effectiveness (${C}_{V}=0.1,\eta =0.9$) (blue curves) increase vaccination rates faster and more sustainably due to better perceived payoffs in all circumstances. In contrast, when the vaccination cost is high, and efficiency is poor (${C}_{V}=0.9,\eta =0.1$) (red curves), uptake is sluggish and plateaus, indicating minimal motivation to vaccinate. Medium circumstances (${C}_{V}=0.5,\eta =0.5$) show considerable uptake on the green curve.

The adaptive approach (top right) shows a gradual increase in testing rates across all conditions, with testing rising significantly more when vaccination is less effective (red), possibly due to a greater perceived risk of infection. Total testing outcomes are higher with the EGT-based testing technique (bottom-right). The red curve (unfavorable vaccine circumstances) has the highest EGT-based testing rate, as people are more likely to test when immunization is ineffective or expensive. Under EGT, the blue curve (favorable vaccine circumstances) shows the lowest testing rate, as effective and widespread immunization reduces the incentive to test. This implies a compensating behavioral mechanism: when vaccination is less desirable, EGT increases testing, whereas favorable vaccination circumstances diminish testing, particularly when both behaviors are strategically directed.

## Conclusion

A comprehensive evaluation of the influence of strategic decision-making, informed by testing and pharmacological vaccination and treatment, on the early detection and ongoing control of infectious disease outbreaks is conducted through extensive numerical simulations. We incorporate evolutionary game theory (EGT) into a dynamic epidemic model to simulate the co-evolution of individual behavior and disease propagation in response to varying costs and benefits of testing, vaccination, and treatment. Results highlight the crucial role of diagnostic testing in facilitating the acceptance of pharmaceuticals. Precise and accessible testing reduces confusion about the disease state, thereby increasing individuals’ willingness to be vaccinated or to pursue treatment. The replicator dynamics indicate that people modify their strategies, testing, vaccinating, or abstaining according to perceived payoffs shaped by the prevalence of illness, the effectiveness of interventions, and related costs. Population dynamics may vary, stabilize, or diverge depending on thresholds for essential parameters.

A crucial conclusion is that effective disease mitigation depends not only on high vaccination effectiveness or treatment coverage, but also on their coordinated execution with timely, adaptive testing procedures. When vaccination uptake is inadequate, even the most efficacious vaccines may be ineffective at preventing large-scale epidemics due to delayed behavioral changes. Conversely, extensive and continuous diagnostic testing expedites case detection and promotes preventive measures, thereby reducing the overall magnitude of the epidemic and improving societal outcomes. Subsidized or free testing early in an epidemic encourages early and frequent testing. Testing, vaccination, and treatment should be integrated into a coordinated public health program so that these interventions operate synergistically rather than as a single control strategy. Policy responses should remain adaptive, with financial incentives and appropriate restrictions adjusted in line with epidemic trends and observed testing behavior. Public communication should clearly emphasize both the individual and collective benefits of testing and preventive treatment in order to strengthen voluntary compliance. Behavior-sensitive delivery strategies, including mobile testing units and app-based alert systems, may further reduce barriers to access, effort, and decision fatigue. In addition, community-based awareness initiatives and culturally tailored communication should be directed toward high-risk and low-compliance populations to improve outreach and participation. Real-time surveillance and feedback systems are also needed to monitor engagement and behavioral responses, enabling public health policies to be refined as conditions evolve. Ultimately, epidemic control measures must combine pharmaceutical interventions with behavioral dynamics to achieve adequate control. A synchronized approach that connects personal incentives with public health objectives will be crucial in reducing the severity of epidemics, ensuring equitable access to healthcare, and fostering long-term societal resilience.

## Figures and Tables

**Fig. 24:**
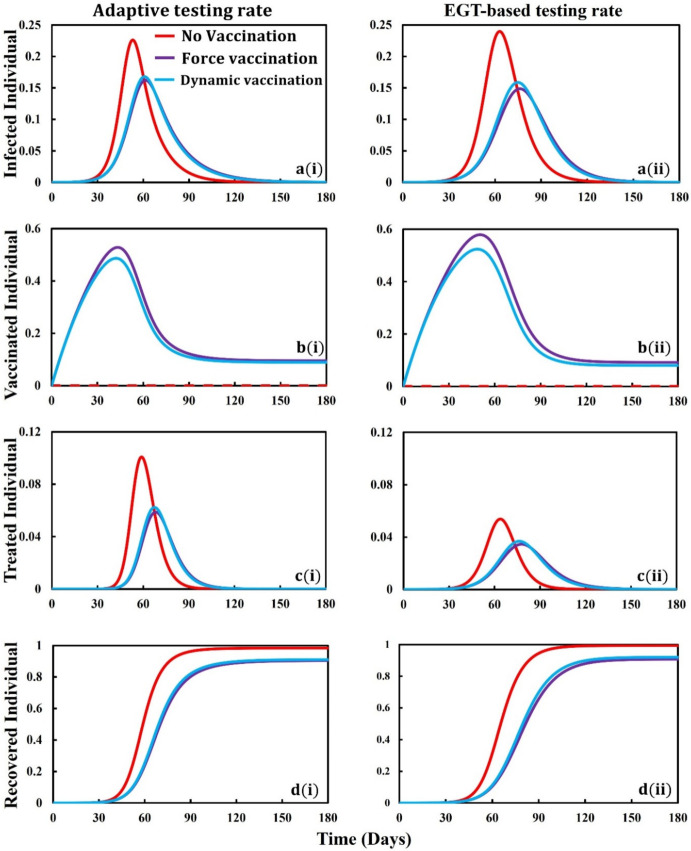
The effect of no vaccination $\left({x}_{0}=0\right)$, force vaccination $\left(x=0.02,{m}_{x}=0\right)$ and dynamic vaccination $\left({x=0.02,m}_{x}=0.1\right)$ (EGT) on the infected, vaccinated, treated, and recovered individuals through adaptive (panel i*-) and EGT-based (panel ii*-) testing rates. The parameter settings are $\beta =0.8333,\gamma =0.1,\alpha =0.2,\phi =0.05,{B}_{V}=0.2,a=0.2,\tau =0.9,\sigma =0.2,\omega =0.2,{m}_{y}=0.1,{C}_{V}=0.5,\eta =0.5,{C}_{y}=0.1.$ For adaptive testing case, $k=0.1,\lambda =0.1,$ and EGT case, ${y}_{M}=0.2,\delta =0.1$ (Color figure online)

**Fig. 25:**
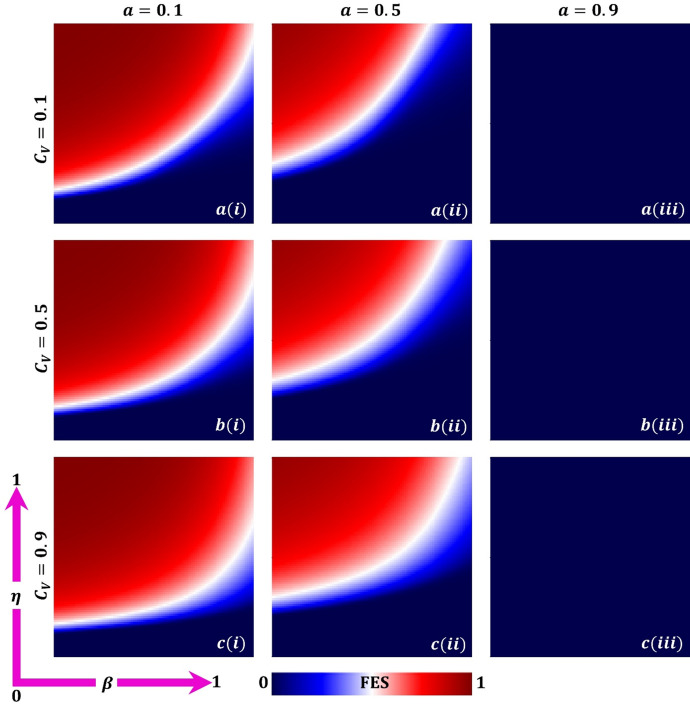
2D graphical representation of the final epidemic size (FES) of disease dynamics based on the vaccination cost $({C}_{V}=\mathrm{0.1,0.5,0.9})$ and personal protection rate $(a=\mathrm{0.1,0.5,0.9})$ with adaptive testing and EGT-based vaccination rates, where the remaining parameter values are as follows: $\alpha =0.2,\gamma =0.1,\phi =0.05,\tau =0.9,\sigma =0.3,\delta =0.1,{m}_{x}=0.1, {B}_{V}=0.2,\omega =0.05,\lambda =0.5,k=0.1,{C}_{y}=0.1 x$-axis specifies the disease transmission rate $(\beta ),$ and the $y$-axis designates vaccine efficacy $(\eta )$ (Color figure online)

**Fig. 26:**
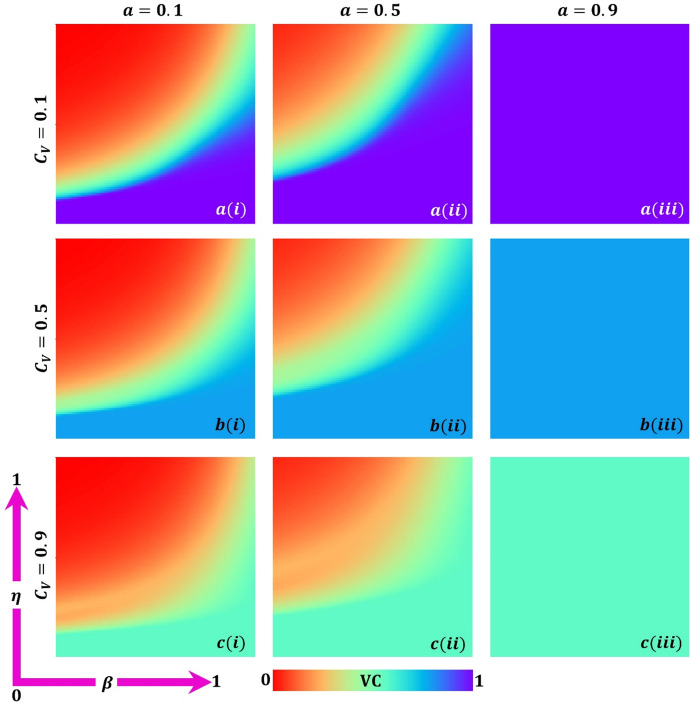
2D graphical representation of vaccination coverage (VC) of disease dynamics based on the vaccination cost $({C}_{V}=\mathrm{0.1,0.5,0.9})$ and personal protection rate $(a=\mathrm{0.1,0.5,0.9})$ with adaptive testing and EGT-based vaccination rates, where the remaining parameter values are as follows: $\alpha =0.2,\gamma =0.1,\phi =0.05,\tau =0.9,\sigma =0.3,\delta =0.1,{m}_{x}=0.1, {B}_{V}=0.2,\omega =0.05,\lambda =0.5,k=0.1,{C}_{y}=0.1$. $x$-axis specifies the disease transmission rate $(\beta ),$ and the $y$-axis designates vaccine efficacy $(\eta )$ (Color figure online)

**Fig. 27:**
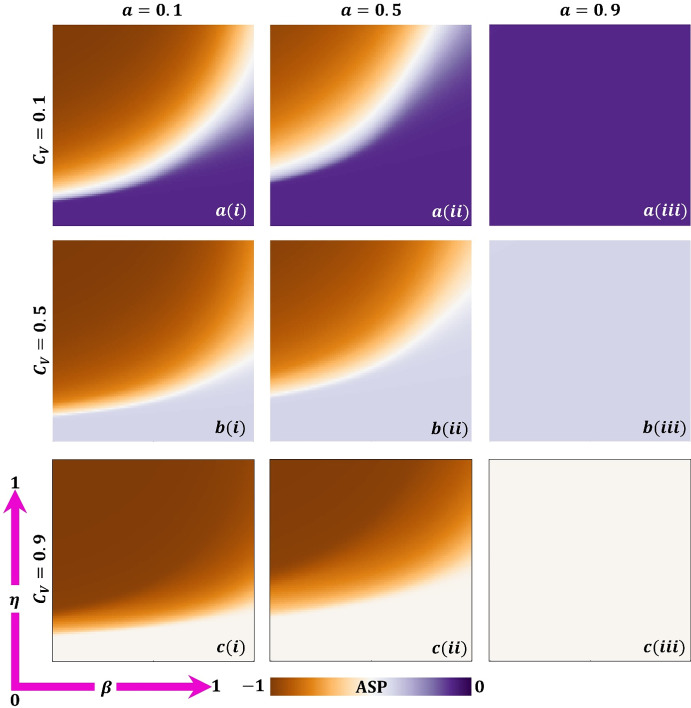
2D graphical representation of average social payoff (ASP) of disease dynamics based on the vaccination cost $({C}_{V}=\mathrm{0.1,0.5,0.9})$ and personal protection rate $(a=\mathrm{0.1,0.5,0.9})$ with adaptive testing and EGT-based vaccination rates, where the remaining parameter values are as follows: $\alpha =0.2,\gamma =0.1,\phi =0.05,\tau =0.9,\sigma =0.3,\delta =0.1,{m}_{x}=0.1, {B}_{V}=0.2,\omega =0.05,\lambda =0.5,k=0.1,{C}_{y}=0.1$. $x$-axis specifies the disease transmission rate $(\beta ),$ and the $y$-axis designates vaccine efficacy $(\eta )$ (Color figure online)

**Fig. 28:**
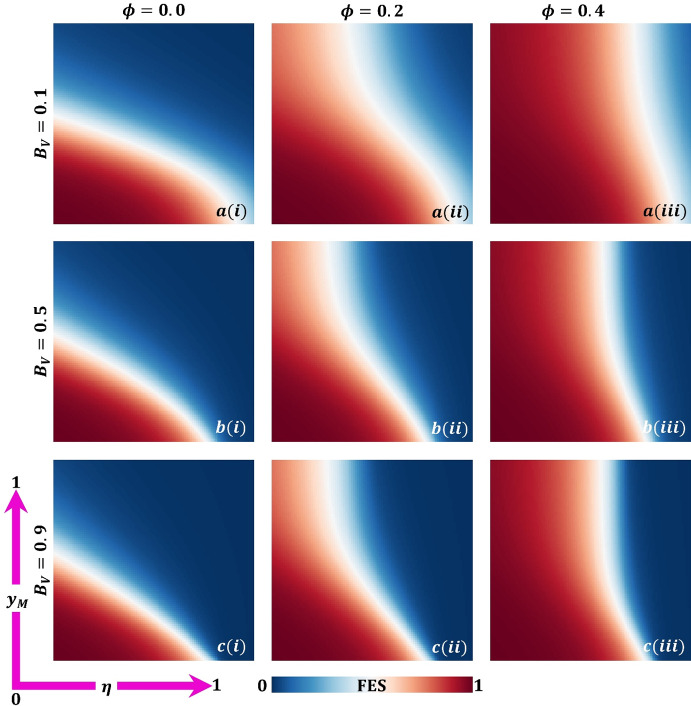
2D graphical representation of the final epidemic size (FES) of disease dynamics based on the benefit of vaccination $({B}_{V}=\mathrm{0.1,0.5,0.9})$ and false negative results rate $(\phi =\mathrm{0.0,0.2,0.4})$ with EGT-based testing and vaccination rates, where the remaining parameter values are as follows: $\beta =0.8333,\alpha =0.2,\gamma =0.1,\tau =0.9,\sigma =0.2,\delta =0.1,{m}_{x}=0.1, {C}_{V}=0.3,{\omega =0.2,m}_{y}=0.1,{C}_{y}=0.1$. $x$-axis designates vaccine efficacy $(\eta ),$ and the $y$-axis specifies the motivation to participate in testing $({y}_{M})$ (Color figure online)

**Fig. 29:**
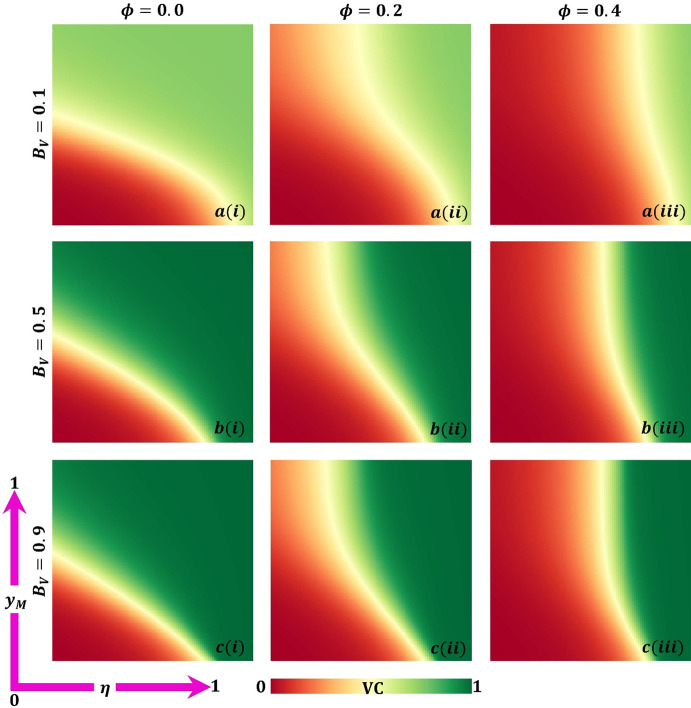
2D graphical representation of vaccination coverage (VC) of disease dynamics based on the benefit of vaccination $({B}_{V}=\mathrm{0.1,0.5,0.9})$ and false negative results rate $(\phi =\mathrm{0.0,0.2,0.4})$ with EGT-based testing and vaccination rates, where the remaining parameter values are as follows: $\beta =0.8333,\alpha =0.2,\gamma =0.1,\tau =0.9,\sigma =0.2,\delta =0.1,{m}_{x}=0.1, {C}_{V}=0.3,{\omega =0.2,m}_{y}=0.1,{C}_{y}=0.1$. $x$-axis designates vaccine efficacy $(\eta ),$ and the $y$-axis specifies the motivation to participate in testing $({y}_{M})$ (Color figure online)

**Fig. 30:**
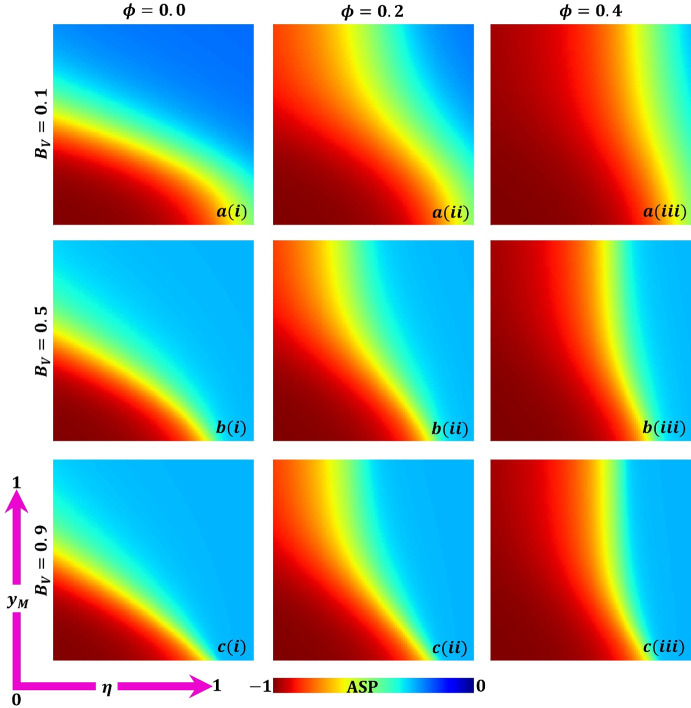
2D graphical representation of average social payoff (ASP) of disease dynamics based on the benefit of vaccination $({B}_{V}=\mathrm{0.1,0.5,0.9})$ and false negative results rate $(\phi =\mathrm{0.0,0.2,0.4})$ with EGT-based testing and vaccination rates, where the remaining parameter values are as follows: $\beta =0.8333,\alpha =0.2,\gamma =0.1,\tau =0.9,\sigma =0.2,\delta =0.1,{m}_{x}=0.1, {C}_{V}=0.3,{\omega =0.2,m}_{y}=0.1,{C}_{y}=0.1$. $x$-axis designates vaccine efficacy $(\eta ),$ and the $y$-axis specifies the motivation to participate in testing $({y}_{M})$

**Fig. 31:**
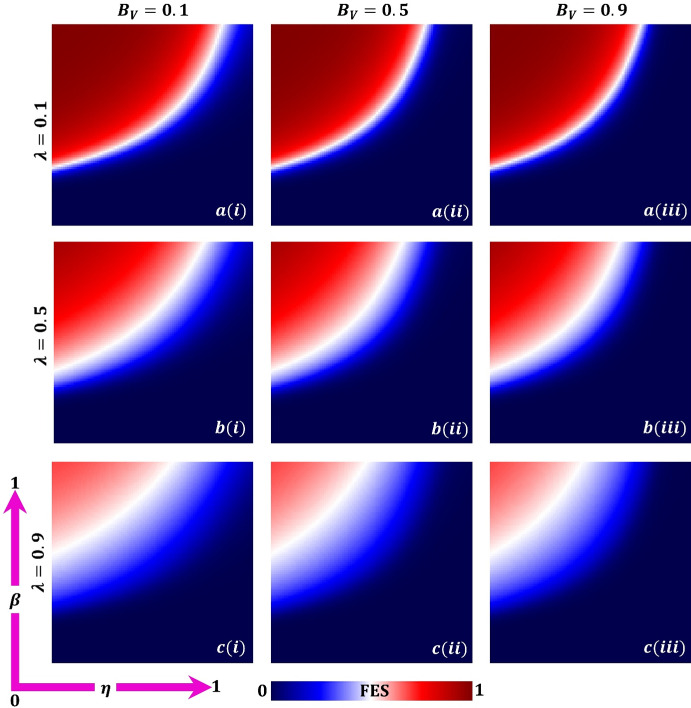
2D graphical representation of the final epidemic size (FES) of disease dynamics based on the benefit of vaccination $({B}_{V}=\mathrm{0.1,0.5,0.9})$ and testing controlling rate $(\lambda =\mathrm{0.1,0.5,0.9})$ with adaptive testing and EGT-based vaccination rates, where the remaining parameter values are as follows: $a=0.3,\alpha =0.2,\gamma =0.1,\phi =0.05,\tau =0.9,\sigma =0.2,\delta =0.1,{m}_{x}=0.1, {C}_{V}=0.1,\omega =0.01,{C}_{y}=0.1,k=0.1$. $x$-axis designates vaccine efficacy $(\eta ),$ and the $y$-axis specifies the disease transmission rate $(\beta )$ (Color figure online)

**Fig. 32:**
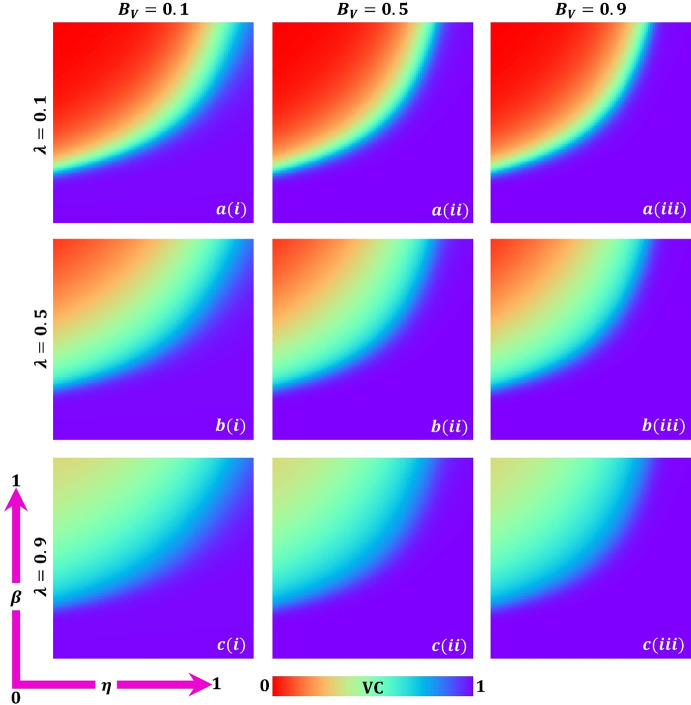
2D graphical representation of vaccination coverage (VC) of disease dynamics based on the benefit of vaccination $({B}_{V}=\mathrm{0.1,0.5,0.9})$ and testing controlling rate $(\lambda =\mathrm{0.1,0.5,0.9})$ with adaptive testing and EGT-based vaccination rates, where the remaining parameter values are as follows: $a=0.3,\alpha =0.2,\gamma =0.1,\phi =0.05,\tau =0.9,\sigma =0.2,\delta =0.1,{m}_{x}=0.1, {C}_{V}=0.1,\omega =0.01,{C}_{y}=0.1,k=0.1$. $x$-axis designates vaccine efficacy $(\eta ),$ and the $y$-axis specifies the disease transmission rate $(\beta )$ (Color figure online)

**Fig. 33:**
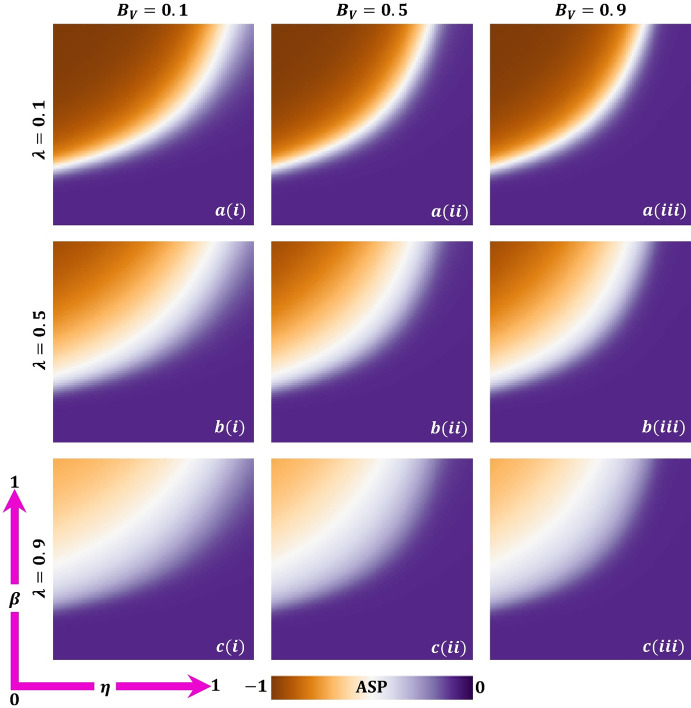
2D graphical representation of average social payoff (ASP) of disease dynamics based on the benefit of vaccination $({B}_{V}=\mathrm{0.1,0.5,0.9})$ and testing controlling rate $(\lambda =\mathrm{0.1,0.5,0}.9)$ with adaptive testing and EGT-based vaccination rates, where the remaining parameter values are as follows: $a=0.3,\alpha =0.2,\gamma =0.1,\phi =0.05,\tau =0.9,\sigma =0.2,\delta =0.1,{m}_{x}=0.1, {C}_{V}=0.1,\omega =0.01,{C}_{y}=0.1,k=0.1$. $x$-axis designates vaccine efficacy $(\eta ),$ and the $y$-axis specifies the disease transmission rate $(\beta )$ (Color figure online)

**Fig. 34:**
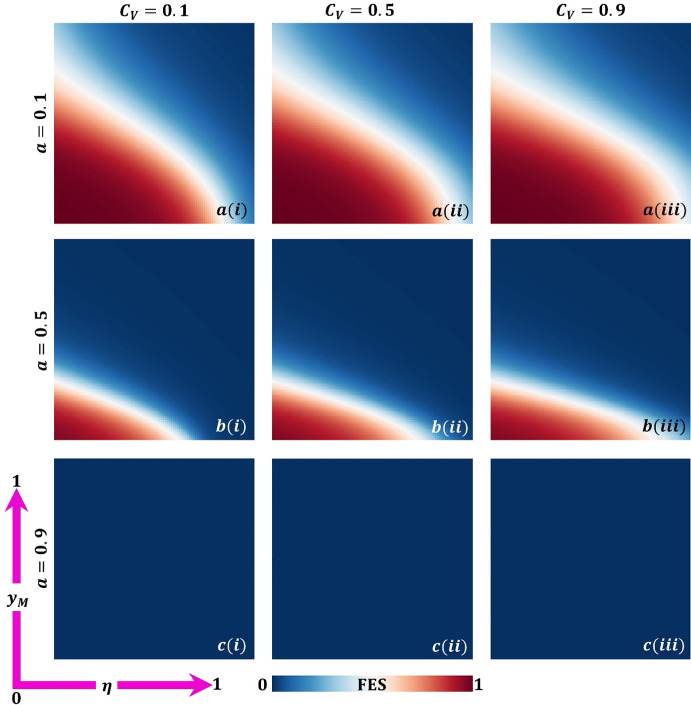
2D graphical representation of final epidemic size (FES) of disease dynamics based on personal awareness rate $(a=\mathrm{0.1,0.5,0.9})$ and cost of vaccination $({C}_{V}=\mathrm{0.1,0.5,0.9})$ with EGT-based testing and vaccination rates, where the remaining parameter values are as follows: $\beta =0.8333,\alpha =0.2,\gamma =0.1,\phi =0.05,\tau =0.9,\sigma =0.2,\delta =0.1,{m}_{x}=0.1, {B}_{V}=0.2,{\omega =0.2,m}_{y}=0.1,{C}_{y}=0.1$. $x$-axis designates vaccine efficacy $(\eta ),$ and the $y$-axis specifies the motivation to participate in testing $({y}_{M})$ (Color figure online)

**Fig. 35:**
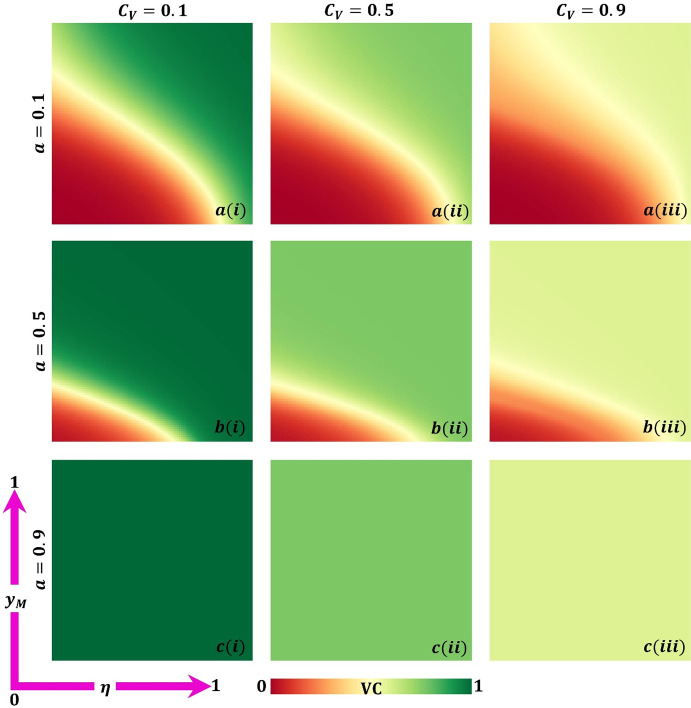
2D graphical representation of vaccination coverage (VC) of disease dynamics based on personal awareness rate $(a=\mathrm{0.1,0.5,0.9})$ and cost of vaccination $({C}_{V}=\mathrm{0.1,0.5,0.9})$ with EGT-based testing and vaccination rates, where the remaining parameter values are as follows: $\beta =0.8333,\alpha =0.2,\gamma =0.1,\phi =0.05,\tau =0.9,\sigma =0.2,\delta =0.1,{m}_{x}=0.1, {B}_{V}=0.2,{\omega =0.2,m}_{y}=0.1,{C}_{y}=0.1$. $x$-axis designates vaccine efficacy $(\eta ),$ and the $y$-axis specifies the motivation to participate in testing $({y}_{M})$ (Color figure online)

**Fig. 36:**
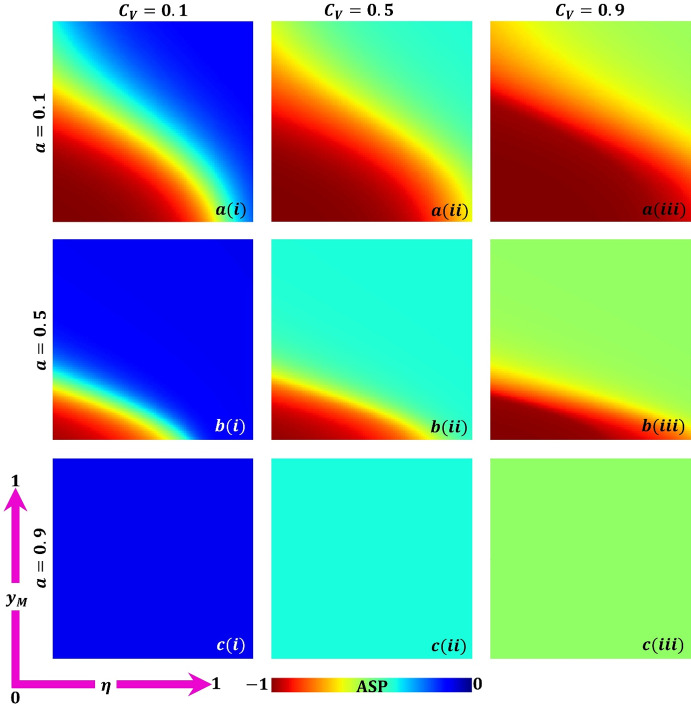
2D graphical representation of average social payoff (ASP) of disease dynamics based on personal awareness rate $(a=\mathrm{0.1,0.5,0.9})$ and cost of vaccination $({C}_{V}=\mathrm{0.1,0.5,0.9})$ with EGT-based testing and vaccination rates, where the remaining parameter values are as follows: $\beta =0.8333,\alpha =0.2,\gamma =0.1,\phi =0.05,\tau =0.9,\sigma =0.2,\delta =0.1,{m}_{x}=0.1, {B}_{V}=0.2,{\omega =0.2,m}_{y}=0.1,{C}_{y}=0.1$. $x$-axis designates vaccine efficacy $(\eta ),$ and the $y$-axis specifies the motivation to participate in testing $({y}_{M})$ (Color figure online)

**Fig. 37:**
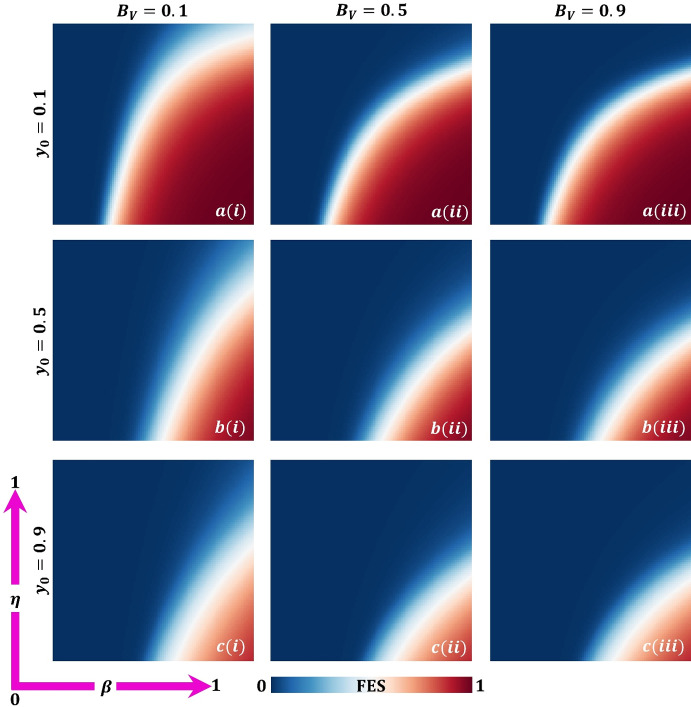
2D graphical representation of the final epidemic size (FES) of disease dynamics based on the benefit of vaccination $({B}_{V}=\mathrm{0.1,0.5,0.9})$ and testing rate $({y}_{0}=\mathrm{0.1,0.5,0.9})$ with EGT-based testing and vaccination rates, where the remaining parameter values are as follows: $a=0.2,\alpha =0.2,\gamma =0.1,\phi =0.05,\tau =0.9,\sigma =0.2,\delta =0.1,{m}_{x}=0.1, {C}_{V}=0.3,{\omega =0.2,m}_{y}=0.1,{C}_{y}=0.1,{y}_{M}=0.2$. $x$-axis specifies the disease transmission rate $(\beta ),$ and the $y$-axis designates vaccine efficacy $(\eta )$ (Color figure online)

**Fig. 38:**
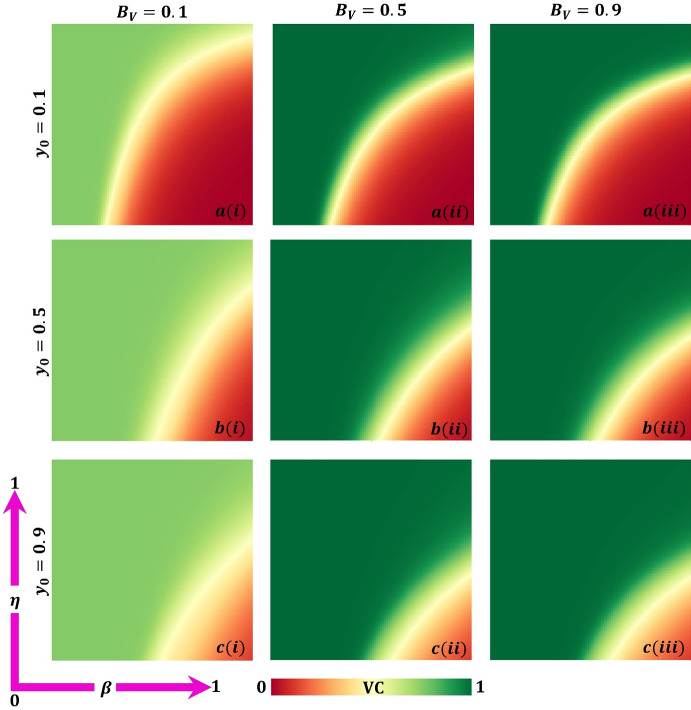
2D graphical representation of vaccination coverage (VC) of disease dynamics based on the benefit of vaccination $({B}_{V}=\mathrm{0.1,0.5,0.9})$ and testing rate $({y}_{0}=\mathrm{0.1,0.5,0.9})$ with EGT-based testing and vaccination rates, where the remaining parameter values are as follows: $a=0.2,\alpha =0.2,\gamma =0.1,\phi =0.05,\tau =0.9,\sigma =0.2,\delta =0.1,{m}_{x}=0.1, {C}_{V}=0.3,{\omega =0.2,m}_{y}=0.1,{C}_{y}=0.1,{y}_{M}=0.2$. $x$-axis specifies the disease transmission rate $(\beta ),$ and the $y$-axis designates vaccine efficacy $(\eta )$ (Color figure online)

**Fig. 39:**
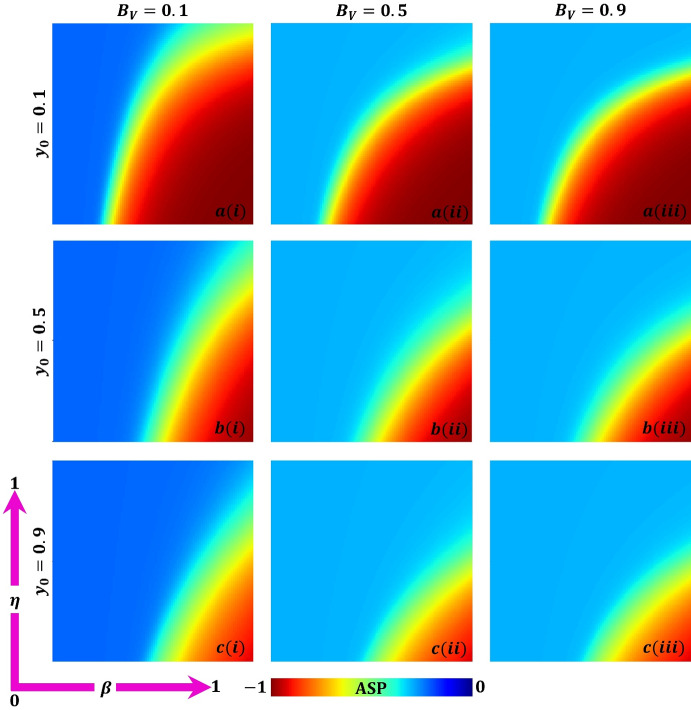
2D graphical representation of average social payoff (ASP) of disease dynamics based on the benefit of vaccination $({B}_{V}=\mathrm{0.1,0.5,0.9})$ and testing rate $({y}_{0}=\mathrm{0.1,0.5,0.9})$ with EGT-based testing and vaccination rates, where the remaining parameter values are as follows: $a=0.2,\alpha =0.2,\gamma =0.1,\phi =0.05,\tau =0.9,\sigma =0.2,\delta =0.1,{m}_{x}=0.1, {C}_{V}=0.3,{\omega =0.2,m}_{y}=0.1,{C}_{y}=0.1,{y}_{M}=0.2$. $x$-axis specifies the disease transmission rate $(\beta ),$ and the $y$-axis designates vaccine efficacy $(\eta )$ (Color figure online)

## Data Availability

The datasets used and/or analyzed during the current study are available from the corresponding author upon reasonable request.

## Appendix

It is worth mentioning that $x$ and $y$ are constant for theoretical analysis.

### Mathematical Analysis of the Model

The proposed testing-based epidemic model (1.1–1.7) has a feasible domain as follows:

3 $$
\begin{aligned}\Pi =\left\{\left(S,V,E,{U}_{I},{D}_{I},T,R\right)\ge 0|S\le \frac{\Lambda }{x+\mu },V\le \frac{\Lambda x}{\mu \left(x+\mu \right)},S+V+E+{U}_{I}+{D}_{I}+T+R\le \frac{\Lambda }{\mu }\right\}.\end{aligned}
$$

It can be readily shown that $\Pi$ is positively invariant for the vector field of the nonlinear system of Eqs. (1.1–1.7). The following conclusion can be readily obtained:

#### Theorem 1

In the feasible domain $\Pi ,$ the proposed model (1.1–1.7) has a unique disease-free equilibrium (DFE) point.

4 $${\mathbb{E}}_{0}=\left({S}^{0},{V}^{0},{E}^{0},{U}_{I}^{0},{D}_{I}^{0},{T}^{0},{R}^{0}\right)=\left(\frac{\Lambda }{x+\mu },\frac{\Lambda x}{\mu (x+\mu )},\mathrm{0,0},\mathrm{0,0},0\right).$$

#### Proof

Recall the model Eq. (1.1–1.7). The disease-free equilibrium point is the point where there is no disease. To obtain the DFE, one must set all disease-related compartments equal to zero. Therefore, assume $E={U}_{I}={D}_{I}=0$ and substitute these values in the model. Then one can write,

$${\mathbb{E}}_{0}=\left({S}^{0},{V}^{0},{E}^{0},{U}_{I}^{0},{D}_{I}^{0},{T}^{0},{R}^{0}\right)=\left(\frac{\Lambda }{x+\mu },\frac{\Lambda x}{\mu (x+\mu )},\mathrm{0,0},\mathrm{0,0},0\right).$$

### Basic reproduction number

The basic reproduction number of our model will be obtained by using the conventional next-generation matrix approach (Driessche and Watmough 2002), using the newly established infection matrix $F$ and the transition matrix $V$ calculated as follows:

$$
 F = \left[ {\begin{array}{*{20}c} 0 & {\beta \left( {1 - a} \right)S^{0} + \beta \left( {1 - \eta } \right)\left( {1 - a} \right)V^{0} } & {\beta \left( {1 - a} \right)S^{0} + \beta \left( {1 - \eta } \right)\left( {1 - a} \right)V^{0} } \\ 0 & 0 & 0 \\ 0 & 0 & 0 \\ \end{array} } \right]\;{\mathrm{and}}\;
$$

$$ V = \left[ {\begin{array}{*{20}c} {\alpha + \mu } & 0 & 0 \\ { - \alpha \left( {1 - y} \right) - \alpha y\phi } & {\gamma + \mu } & 0 \\ { - \alpha y\left( {1 - \phi } \right)} & 0 & {\tau + \gamma + \mu } \\ \end{array} } \right] $$

Thus, the basic reproduction number, *R*_0_ is the largest Eigenvalue of$\left| {FV^{ - 1} } \right|$, as follows,

5 $$
\begin{aligned}
R_{0} &= \alpha \left\{ {\beta \left( {1 - a} \right)\frac{\Lambda }{x + \mu } + \beta \left( {1 - \eta } \right)\left( {1 - a} \right)\frac{\Lambda x}{{\mu \left( {x + \mu } \right)}}} \right\}\\
&\quad\left[ {\frac{1 - y + y\varphi }{{\left( {\alpha + \mu } \right)\left( {\gamma + \mu } \right)}} + \frac{{y\left( {1 - \varphi } \right)}}{{\left( {\alpha + \mu } \right)\left( {\gamma + \mu + \tau } \right)}}} \right].
\end{aligned}
$$

#### Theorem 2

In the feasible domain $\Pi ,$ the proposed model (1.1–1.7) has a positive endemic equilibrium (EE) point.

$${\mathbb{E}}_{*}=\left({S}^{*},{V}^{*},{E}^{*},{U}_{I}^{*},{{D}_{I}^{*},{T}^{*},R}^{*}\right)$$

for ${R}_{0}>1$ and unique when it exists.

#### Proof

One can write the nontrivial equilibrium of the nonlinear system (1.1–1.7) as follows,

$$ 0 = {\Lambda } - \beta \left( {1 - a} \right)\left( {U_{I} + D_{I} } \right)S - \left( {x + \mu } \right)S, $$

$$ 0 = xS - \beta \left( {1 - \eta } \right)\left( {U_{I} + D_{I} } \right)V - \mu V $$

$$0=\beta (1-a)\left({U}_{I}+{D}_{I}\right)S+\beta (1-\eta )(1-a)({U}_{I}+{D}_{I})V-(\alpha +\mu )E,$$

$$0=\alpha \left(1-y\right)E+\alpha y\phi E-(\gamma +\mu ){U}_{I},$$

$$0=\alpha y\left(1-\phi \right)E-\left(\tau +\gamma +\mu \right){D}_{I},$$

$$0=\tau {D}_{I}-(\sigma +\mu )T,$$

$$0=\gamma \left({U}_{I}+{D}_{I}\right)+\sigma T-\mu R.$$

Now, one can write,

6 $$S=\frac{\Lambda }{\beta \left(1-a\right)\left({U}_{I}+{D}_{I}\right)+(x+\mu )}, $$

$$V=\frac{xS}{\beta \left(1-\eta \right)\left(1-a\right)\left({U}_{I}+{D}_{I}\right)+\mu }.$$

Then

7 $$V=\frac{x\Lambda }{\left\{\beta \left(1-\eta \right)\left(1-a\right)\left({U}_{I}+{D}_{I}\right)+\mu \right\}\left\{\beta \left(1-a\right)\left({U}_{I}+{D}_{I}\right)+\left(x+\mu \right)\right\}}, $$

8 $$E=\frac{\beta \left(1-a\right)\left({U}_{I}+{D}_{I}\right)S+\beta \left(1-\eta \right)\left(1-a\right)\left({U}_{I}+{D}_{I}\right)V}{\alpha +\mu }, $$

9 $${U}_{I}=\frac{\alpha \left(1-y\right)E+\alpha y\phi E}{\gamma +\mu }, $$

10 $${D}_{I}=\frac{\alpha y\left(1-\phi \right)E}{\tau +\gamma +\mu }, $$

From Eqs. (9) and (10), we have

$$
{U}_{I}+{D}_{I}=\frac{\alpha \left(1-y\right)E+\alpha y\phi E}{\gamma +\mu }+\frac{\alpha y\left(1-\phi \right)E}{\tau +\gamma +\mu }=\left\{\frac{\alpha \left(1-y\right)+\alpha y\phi }{\gamma +\mu }+\frac{\alpha y\left(1-\phi \right)}{\tau +\gamma +\mu }\right\}E
$$

11 $$\Rightarrow E=\frac{{U}_{I}+{D}_{I}}{\frac{\alpha \left(1-y\right)+\alpha y\phi }{\gamma +\mu }+\frac{\alpha y\left(1-\phi \right)}{\tau +\gamma +\mu }}. $$

Now, equating Eqs. (8) and (11), we obtain

$$\frac{{U}_{I}+{D}_{I}}{\frac{\alpha \left(1-y\right)+\alpha y\phi }{\gamma +\mu }+\frac{\alpha y\left(1-\phi \right)}{\tau +\gamma +\mu }}=\frac{\beta \left(1-a\right)\left({U}_{I}+{D}_{I}\right)S+\beta \left(1-\eta \right)\left(1-a\right)\left({U}_{I}+{D}_{I}\right)V}{\alpha +\mu }$$

$$\Rightarrow \frac{1}{\frac{\alpha \left(1-y\right)+\alpha y\phi }{\gamma +\mu }+\frac{\alpha y\left(1-\phi \right)}{\tau +\gamma +\mu }}=\frac{\beta \left(1-a\right)S+\beta \left(1-\eta \right)\left(1-a\right)V}{\alpha +\mu }$$

$$\Rightarrow \frac{1}{\frac{\alpha \left(1-y\right)+\alpha y\phi }{\gamma +\mu }+\frac{\alpha y\left(1-\phi \right)}{\tau +\gamma +\mu }}=\frac{\beta \left(1-a\right)S}{\alpha +\mu }+\frac{\beta \left(1-\eta \right)\left(1-a\right)V}{\alpha +\mu }$$

$$
\begin{aligned}
\Rightarrow \frac{1}{\frac{\alpha \left(1-y\right)+\alpha y\phi }{\gamma +\mu }+\frac{\alpha y\left(1-\phi \right)}{\tau +\gamma +\mu }}&=\frac{\beta \left(1-a\right)\Lambda }{(\alpha +\mu )\{\beta \left(1-a\right)\left({U}_{I}+{D}_{I}\right)+(x+\mu )\}}\\
&+\frac{\beta \left(1-\eta \right)\left(1-a\right)x\Lambda }{(\alpha +\mu )\left\{\beta \left(1-\eta \right)\left(1-a\right)\left({U}_{I}+{D}_{I}\right)+\mu \right\}\{\beta \left(1-a\right)\left({U}_{I}+{D}_{I}\right)+(x+\mu )\}}
\end{aligned}
$$

12 $$
\begin{aligned}
&\Rightarrow \frac{\left(\gamma +\mu \right)\left(\tau +\gamma +\mu \right)}{\left(\tau +\gamma +\mu \right)\left\{\alpha \left(1-y\right)+\alpha y\phi \right\}+\alpha y\left(1-\phi \right)\left(\gamma +\mu \right)} \\
&\quad = \frac{\beta \left(1-a\right)\Lambda }{(\alpha +\mu )\{\beta \left(1-a\right)I+(x+\mu )\}} \\
&\quad + \frac{\beta \left(1-\eta \right)\left(1-a\right)x\Lambda }{(\alpha +\mu )\left\{\beta \left(1-\eta \right)\left(1-a\right)I+\mu \right\}\{\beta \left(1-a\right)I+(x+\mu )\}} .
\end{aligned}
$$

For simplicity, here we assume the total infection of the proposed epidemic model is $I$, i.e., $I={U}_{I}+{D}_{I}.$

Therefore, we are now able to define an equation in terms of a single variable $I$ as follows:

13 $$ f\left( I \right) = \frac{{\left( {\gamma + \mu } \right)\left( {\tau + \gamma + \mu } \right)}}{{\left( {\tau + \gamma + \mu } \right)\left\{ {\alpha \left( {1 - y} \right) + \alpha y\phi } \right\} + \alpha y\left( {1 - \phi } \right)\left( {\gamma + \mu } \right)}}. $$

Hence,

14 $$
\begin{aligned}
f\left( I \right) &= \frac{{\beta \left( {1 - a} \right){\Lambda }}}{{\left( {\alpha + \mu } \right)\left\{ {\beta \left( {1 - a} \right)I + \left( {x + \mu } \right)} \right\}}} \\
&\quad + \frac{{\beta \left( {1 - \eta } \right)\left( {1 - a} \right)x{\Lambda }}}{{\left( {\alpha + \mu } \right)\left\{ {\beta \left( {1 - \eta } \right)\left( {1 - a} \right)I + \mu } \right\}\left\{ {\beta \left( {1 - a} \right)I + \left( {x + \mu } \right)} \right\}}}.
\end{aligned}
$$

Clearly, when $I>0$, the function $f\left(I\right)$ is strictly decreasing. Therefore, when $I\to \infty ,$ $f\left(I\right)>0.$ Thus, we can conclude that at $I={I}^{*}>0,$ the Eq. (13) has a positive solution if and only if,

15 $$f\left(0\right)>\frac{\left(\gamma +\mu \right)\left(\tau +\gamma +\mu \right)}{\left(\tau +\gamma +\mu \right)\left\{\alpha \left(1-y\right)+\alpha y\phi \right\}+\alpha y\left(1-\phi \right)\left(\gamma +\mu \right)}. $$

Therefore, the above Eq. (15) is equivalent to ${R}_{0}>1$, an indicator of outbreaks by use of elementary algebraic manipulation. Hence, by the monotonicity properties of the function $f$, ${I}^{*}$ is unique. Analogously, based on the earlier-mentioned equations, it is easy to determine $S,V,E, T,$ and $R$ uniquely at the positive equilibrium of ${I}^{*}.$ In recapitulation, a unique endemic equilibrium point ${\mathbb{E}}_{*}=\left({S}^{*}, {V}^{*},{E}^{*},{U}_{I}^{*},{D}_{I}^{*},{T}^{*},{R}^{*}\right)$ exists for the system of nonlinear Eqs. (1.1–1.7) if and only if ${R}_{0}>1.$

## Definition of FES, VC, and ASP

### Final Epidemic Size (FES)

The final epidemic size (FES) is a fundamental outcome measure in infectious disease modeling, representing the cumulative number or proportion of individuals who become infected over the entire course of an epidemic. In deterministic compartmental models, the FES is usually obtained by integrating the governing differential equations until the infectious classes approach zero or the system reaches its long-term state. Thus, FES provides an aggregate measure of epidemic burden by quantifying the total extent of disease spread within a population. A smaller FES indicates more effective disease control, whereas a larger FES reflects broader transmission and a greater public health burden (Ouardani et al. 2026; Ullah and Wang 2025).

## Vaccination Coverage (VC)

Vaccination coverage (VC) measures the proportion of the population that has received vaccination against a given disease, an important epidemiological indicator because it reflects the degree of population-level protection and the potential reduction in susceptible individuals. In the proposed model, vaccination coverage is represented by the vaccinated compartment, which contains individuals with vaccine-induced protection. Increasing VC reduces the FES by lowering the number of susceptible individuals available for infection. However, the effect of vaccination depends on vaccine efficacy, the timing of vaccination, and the overall level of population coverage. When vaccination coverage is sufficiently high, indirect protection may also arise, reducing transmission even among unvaccinated individuals. Conversely, inadequate vaccination coverage can sustain transmission, leading to a larger epidemic and potentially more severe public-health consequences (Ouardani et al. 2026; Ullah and Wang 2025).

## Average Social Payoff (ASP)

The average social payoff (ASP) is a game-theoretic measure used to evaluate the collective benefit or cost associated with behavioral strategies and intervention decisions. In epidemic models with evolutionary game dynamics, ASP summarizes the net population-level outcome resulting from individual choices, such as vaccination, testing, isolation, or treatment-seeking. It reflects the balance between the epidemiological benefits of disease prevention and the perceived costs associated with adopting control measures. In the present model, the average social payoff is used to assess the social performance of different behavioral strategies under epidemic risk. A higher ASP indicates that the population obtains greater net benefit from the adopted intervention strategy, whereas a lower ASP suggests greater social cost, inefficient behavior, or insufficient disease-control effort. Therefore, ASP provides a useful link between epidemiological outcomes and behavioral decision-making, helping to evaluate whether individual strategic choices align with socially desirable outcomes in epidemic control (Ouardani et al. 2026; Ullah and Wang 2025). Mathematically,

$$AS{P}^{NE}=-{C}_{V}{\int}_{0}^{\infty }V\left(v\right)-{\int}_{0}^{\infty }\left({{U}_{I}\left(v\right)+D}_{I}\left(v\right)\right)dv.$$

Here, $AS{P}^{NE}$ denotes the ASP evaluated at the Nash equilibrium (NE), where the vaccination game (intervention game, IG) has reached a locally stable strategic state over the behavioral time scale.

## Additional Results And Discussion

The existence or absence of vaccines ($x=0$), forced vaccination ($x=0.02,{m}_{x}=0.0$), and dynamic vaccination (EGT) strategies significantly influence disease dynamics when analyzed within the framework of adaptive and evolutionary game-theoretic testing rates (Fig. 24). Without a vaccine, the population remains vulnerable, and disease control primarily depends on testing and treatment. Although adaptive testing mitigates infections by swiftly detecting and isolating individuals, the absence of vaccination constrains long-term containment, often leading to ongoing transmission. In the no-vaccination game scenario, where vaccination is accessible but people neglect strategic decision-making, the vaccination rate remains constant or is externally mandated, possibly resulting in inadequate coverage if individuals are apathetic or uninformed. In this context, adaptive testing continues to reduce transmission; however, disease control is still less effective. Conversely, in vaccination games, individuals formulate strategic vaccination choices influenced by perceived infection risk, vaccine cost, and social factors, as outlined by evolutionary game theory (EGT). When integrated with EGT-based testing rates, this setting establishes a dynamic feedback loop: elevated testing uncovers more infected individuals, heightening perceived danger and incentivizing more vaccination, which subsequently reduces transmission. This interaction leads to more efficient and adaptable disease control, aligning individual behavior with public health outcomes. Consequently, vaccination tactics, especially when integrated with adaptive or evolutionary game theory-based testing methodologies, represent the most responsive and effective means of regulating epidemics.

This trio (Figs. 25, 26, 27) of two-dimensional heatmap panels depicts the relationship between vaccination cost $({C}_{V}=0.1, 0.5, 0.9)$ and awareness level $(a= 0.1, 0.5, 0.9)$ concerning the FES, VC, and ASP within a vaccination framework based on adaptive testing and an EGT-based vaccination game. The heatmaps illustrate the transmission rate ($\beta$, $x$-axis) and vaccine efficacy ($\eta$, $y$-axis), offering a detailed perspective on the interplay between behavioral and epidemiological factors in shaping epidemic outcomes. Blue areas in the FES panel (Fig. 28) signify successful control (minimal epidemic size), while red indicates a substantial epidemic burden. As vaccination effectiveness improves and costs diminish, the red region decreases, particularly with heightened awareness. At peak awareness $(a =0.9)$, the FES approaches zero across all scenarios, indicating that the individual’s effective awareness strategy mitigates epidemics when paired with effective immunizations. Again, the 2D heatmap of VC, illustrated in Fig. 26, emphasizes that vaccine adoption escalates with heightened awareness and effectiveness, especially when vaccination costs are low. Low $\eta$ and high ${C}_{V}$ inhibit uptake (as shown by increased red); however, heightened awareness continually propels vaccination coverage toward its apex, irrespective of cost, illustrating the preeminent role of public health facts in vaccination behavior. The purple regions of Fig. 27 signify elevated social payoffs (near 0), while orange denotes diminished payoffs (around $-1$). Analogous to vaccination coverage, societal benefit is maximized when vaccine costs are minimal, effectiveness is elevated, and awareness is robust. Conversely, ASP diminishes sharply with low awareness, high ${C}_{V}$, or a lower value of $\eta$, indicating a compounded public health and economic cost. Ultimately, high awareness, effective vaccinations, and affordability form a synergistic trinity that reduces the magnitude of epidemics, encourages global vaccine uptake, and enhances societal benefits. In this context, adaptive testing enhances these benefits by dynamically adjusting behavioral responses based on disease prevalence and individual perceptions.

Figures 29, 30, 31 display 2D heatmaps of proposed models’ disease dynamics depicting FES, VC, and ASP as a function of vaccine efficacy $(\eta )$ and awareness or motivation of testing participation rate $({y}_{M})$, under different values of vaccination benefit $({B}_{V})$ and false negative results rate $(\phi )$ within an evolutionary game theory (EGT)-based epidemic model. Each subfigure in Fig. 29 illustrates the interactions among various behavioral and testing factors that influence disease outcomes. As $\phi$ increases from 0.0 to 0.4, the efficacy of testing diminishes due to a rise in undetected infections, expanding the red areas indicative of high FES. Advancing along the rows, an increase in ${B}_{V}$ (from 0.1 to 0.9) promotes vaccination, thereby transitioning the system towards diminished epidemic magnitudes, as seen by the increasing blue areas (disease-free equilibrium). The figure highlights the importance of behavioral incentives and testing reliability in epidemic control: inadequate testing (high $\phi$) can hinder control efforts, whereas the robust benefit of vaccination $({B}_{V})$ can mitigate this impact when paired with high vaccine efficacy and adequate public engagement in testing. In addition, Fig. 30 indicates that increased vaccine uptake is correlated with elevated ${B}_{V}$, particularly when both $\eta$ and ${y}_{M}$ are substantial. Concurrently, the 2D heatmap of ASP (Fig. 31) shows that enhanced social payoffs occur in regions with elevated vaccination effectiveness and testing participation, particularly when testing reliability is high (i.e., low $\phi$). Therefore, increasing vaccination benefits and reducing testing errors collectively improve health outcomes, increase vaccine uptake, and enhance overall societal welfare in epidemics influenced by evolutionary game theory.

Figures 32, 33, 34 display the influence of the benefit of vaccination $({B}_{V}=0.1, 0.5, 0.9)$ and the control of the testing rate $\left(\lambda =0.1, 0.5, 0.9\right)$ on three crucial epidemic outcomes: FES, VC, and ASP. Each subfigure plots vaccine efficacy ($\eta$, $x$-axis) against disease transmission rate ($\beta$, $y$-axis), incorporating a constant awareness level and adaptive testing dynamics within an EGT-based vaccination rate framework. In Fig. 32, the FES shows red regions indicating elevated epidemic loads and blue areas suggesting successful epidemic suppression. The outcomes indicate that augmenting $\lambda$ and $\eta$ results in markedly reduced FES, particularly when ${B}_{V}$ is elevated. Despite elevated transmissibility $(\beta )$, robust adaptive testing and significant vaccination effectiveness effectively mitigate the epidemic. Figure 33 illustrates the variation in vaccine uptake under identical circumstances, with hues ranging from purple to blue indicating increased coverage. As anticipated, elevated values of $\eta , \lambda$, and ${B}_{V}$ individually and together augment VC. At a low rate of ${B}_{V}$ vaccination is infrequent over most of the domain; however, as benefits escalate, adaptive testing $(\lambda )$ induces behavioral changes that increase the vaccinated population. In the ASP panel of Fig. 34, purple hues signify elevated social reward (nearer to 0), while orange indicates diminished value (approaching $-1$). The social benefit significantly enhances with a higher $\lambda$, mainly due to its effectiveness in reducing infections via early identification and behavioral reinforcement, which is further enhanced by elevated ${B}_{V}$ levels, which make vaccination more attractive and result in broader adherence. Collectively, these three panels highlight that adaptive testing, when paired with substantial vaccine benefit and efficacy, creates a self-perpetuating feedback loop of protection: it lowers disease burden (FES), encourages widespread vaccination (VC), and improves overall societal benefit (ASP). These results underscore the importance of dynamic testing strategies and behavioral incentives in facilitating effective epidemic control.

The illustration (Figs. 35, 25, 36) presents a series of 2D heatmaps depicting the disease dynamics through FES, VC, and ASP as a function of vaccine efficacy ($\eta$) and awareness or motivation rate of testing participation (${y}_{M}$), across different personal protection or awareness rates ($a=0.1, 0.5, 0.9$) and vaccination costs (${C}_{V}=0.1, 0.5, 0.9$) within the framework of an evolutionary game theory (EGT)-based epidemic model, with vaccine efficacy ($\eta$) represented on the $x$-axis and testing participation awareness rate (${y}_{M}$) on the y-axis. Each row of Fig. 35 represents a progressive public awareness $a$, whereas each column denotes the increasing vaccine cost ${C}_{V}$. The color gradient, transitioning from blue (low FES) to red (high FES), illustrates the influence of these characteristics on epidemic outcomes. At minimal awareness ($a=0.1$, top row), the FES is considerably higher, particularly when $\eta$ and ${y}_{M}$ are low, or the vaccine cost is substantial, indicating that a lower rate of awareness results in ineffective epidemic control. As awareness heightens (middle and lower rows), the blue patches expand markedly, indicating that enhanced individual awareness or personal protection against disease substantially reduces disease transmission, irrespective of vaccination costs. At the peak degree of awareness ($a=0.9$, bottom row), the FES approaches zero across all scenarios, indicating that a sophisticated awareness rate can facilitate extensive vaccination adoption and successful control, even in high ${C}_{V}$. Therefore, awareness is vital in behavioral decision-making, effectively offsetting cost-related obstacles and bolstering successful epidemic control strategies.

In Fig. 25, the color gradient, ranging from red (indicating low vaccination coverage) to green (representing high vaccine coverage), illustrates the degree of population-wide vaccine adoption within an EGT framework for epidemic control. Vaccination coverage is constrained in the uppermost row ($a=0.1$), characterized by lower protection or awareness rates, particularly when vaccination costs are high, suggesting that people hesitate to vaccinate without substantial awareness or incentives. Conversely, the second row ($a=0.5$) shows a significant proliferation of green regions, indicating enhanced vaccination use despite the intensification in ${C}_{V}$. At the peak level of awareness rate of individuals ($a=0.9$), vaccination coverage nears saturation ($VC\approx 1$) when the vaccination cost ${C}_{V}$ is low, particularly when $\eta$ and ${y}_{M}$ are significantly elevated, despite considerable vaccine costs, suggesting that awareness or personal protection rates are pivotal in overcoming cost barriers and fostering widespread vaccination, thereby facilitating more effective disease control via behavior-driven techniques.

Figure 36 encapsulates the comprehensive advantage to the populace from strategic decisions about testing and vaccination, integrating health outcomes and economic factors within an evolutionary game theory (EGT) framework. The color gradient ranges from red (minimum ASP, $-1$) to blue (maximum ASP, 0), signifying that higher values (approaching blue) are socially advantageous. When awareness is minimal (*a* = 0.1, top row), ASP exhibits significant sensitivity to vaccine costs, remaining reasonably elevated only at low ${C}_{V}$, and decreases precipitously with increasing costs. As awareness increases (*a* = 0.5 and *a* = 0.9, center and bottom rows), the ASP upsurges, particularly in contexts with high $\eta$. At the peak level of personal protection to keep individuals safe from disease ($a=0.9$), the social payoff stabilizes across the entire plane, even with elevated vaccination costs, due to extensive vaccine uptake and efficient epidemic control. This figure highlights that personal protection is the primary component in maximizing societal benefit, successfully alleviating the adverse effects of vaccination cost (high ${C}_{V}$) and enabling robust epidemic mitigation techniques from a public safety standpoint.

Figures 37, 38, 39 present the impact of the vaccination benefit (${B}_{V}=0.1, 0.5, 0.9$) and initial testing rate (${y}_{0}=0.1, 0.5, 0.9$) on the FES, VC, and ASP within an evolutionary game theory (EGT)-based framework for testing and vaccination. Each panel plots disease transmission rate ($\beta$, $x$-axis) against vaccine efficacy ($\eta$, $y$-axis), illustrating the combined impact of behavioral and epidemiological characteristics on epidemic outcomes. Blue signifies a minimal final epidemic size in the FES panel (Fig. 37), while red denotes a more extensive outbreak. Augmenting either ${B}_{V}$ or ${y}_{0}$ enlarges the blue area inside the ($\beta , \eta$) space, indicating that an elevated perceived vaccination benefit and more proactive testing contribute to enhanced epidemic suppression. At elevated ${y}_{0}=0.9$ and ${B}_{V}=0.9$, FES remains consistently low across almost all transmission and efficacy levels, affirming its collaborative function in reducing disease burden. The VC panel of Fig. 38 displays vaccination uptake levels (green = high, red = low). Increased testing rates and vaccination advantages expand the region of elevated vaccine coverage, particularly at lower transmission levels and higher vaccine effectiveness. Despite lower effectiveness, robust ${B}_{V}$ or ${y}_{0}$ facilitates extensive vaccination. The red zones (low VC) narrow as either parameter increases, highlighting the behavioral drive arising from individual and group incentives. In the ASP panel (Fig. 39), which quantifies net social gain (ranging from $-1$ to 0), blue areas indicate higher social well-being, while red areas denote significant detriments resulting from disease and inadequate vaccine uptake. The ASP significantly enhances when ${B}_{V}$ and ${y}_{0}$ rise, with optimal results seen in regions where testing is prevalent and vaccination is highly esteemed. Finally, Figs. 37, 38, 39 substantiate that behavioral interventions, which enhance vaccination incentives and augment early testing rates, are essential for achieving a minimal epidemic size, increasing vaccine uptake, and enhancing collective social benefits. These observations highlight the importance of policy strategies that enhance individual motivation and facilitate population-level disease surveillance.
